# Prevalence of internalizing disorders, symptoms, and traits across age using advanced nonlinear models

**DOI:** 10.1017/S0033291721001148

**Published:** 2023-01

**Authors:** Hanna M. van Loo, Lian Beijers, Martijn Wieling, Trynke R. de Jong, Robert A. Schoevers, Kenneth S. Kendler

**Affiliations:** 1 Department of Psychiatry, University of Groningen, University Medical Center Groningen, Groningen, The Netherlands; 2 Department of Information Science, University of Groningen, Groningen, The Netherlands; 3 Lifelines Cohort & Biobank, Roden, The Netherlands; 4 University of Groningen, University Medical Center Groningen, Research School of Behavioural and Cognitive Neurosciences (BCN), Groningen, The Netherlands; 5 Virginia Institute for Psychiatric and Behavioral Genetics, Virginia Commonwealth University, Richmond, VA, USA

**Keywords:** internalizing disorders, neuroticism, negative affect, prevalence, age and sex differences

## Abstract

**Background:**

Most epidemiological studies show a decrease of internalizing disorders at older ages, but it is unclear how the prevalence exactly changes with age, and whether there are different patterns for internalizing symptoms and traits, and for men and women. This study investigates the impact of age and sex on the point prevalence across different mood and anxiety disorders, internalizing symptoms, and neuroticism.

**Methods:**

We used cross-sectional data on 146 315 subjects, aged 18–80 years, from the Lifelines Cohort Study, a Dutch general population sample. Between 2012 and 2016, five current internalizing disorders – major depression, dysthymia, generalized anxiety disorder, social phobia, and panic disorder – were assessed according to DSM-IV criteria. Depressive symptoms, anxiety symptoms, neuroticism, and negative affect (NA) were also measured. Generalized additive models were used to identify nonlinear patterns across age, and to investigate sex differences.

**Results:**

The point prevalence of internalizing disorders generally increased between the ages of 18 and 30 years, stabilized between 30 and 50, and decreased after age 50. The patterns of internalizing symptoms and traits were different. NA and neuroticism gradually decreased after age 18. Women reported more internalizing disorders than men, but the relative difference remained stable across age (relative risk ~1.7).

**Conclusions:**

The point prevalence of internalizing disorders was typically highest between age 30 and 50, but there were differences between the disorders, which could indicate differences in etiology. The relative gap between the sexes remained similar across age, suggesting that changes in sex hormones around the menopause do not significantly influence women's risk of internalizing disorders.

## Introduction

Depressive and anxiety disorders occur across all age ranges and are associated with significant disability (Ferrari et al., 2013; Whiteford et al., 2013). Yet, how exactly internalizing disorders differ across age and sex is a subject of debate and few studies have been able to study their patterns over lifetime in detail. More insight into these patterns can be used to identify target populations for public health interventions (Twenge, Cooper, Joiner, Duffy, & Binau, 2019). Furthermore, this insight could inform hypotheses on specific risk factors for internalizing disorders over the course of life. For example, it has been suggested that changes in women's reproductive hormones during the menopause increase their risk for internalizing disorders, but results are inconclusive (Bryant, Judd, & Hickey, 2012; Judd, Hickey, & Bryant, 2012; Kuehner, 2017; Rössler, Ajdacic-Gross, Riecher-Rössler, Angst, & Hengartner, 2016; Vivian-Taylor & Hickey, 2014). Different developments in prevalence in men and women around the age of menopause could support this hypothesis.

The first question concerns the exact development of different internalizing disorders over lifetime. Most studies in the general population find a decrease of internalizing disorders in older age (de Graaf, ten Have, van Gool, & van Dorsselaer, 2012; Jorm, 2000; Kessler et al., 2010b; Scott et al., 2008; Trollor, Sachdev, Anderson, Andrews, & Brodaty, 2007; Wells et al., 2006). However, it remains unclear whether this decrease in prevalence is linear or nonlinear, and even though it is possible that there are multiple peaks and valleys over the lifetime, most studies use models that cannot identify patterns more complex than a U-curve (Jorm, 2000).

Second, there is a clear gap in the prevalence of depression and anxiety disorders between men and women, with women being affected roughly twice as often as men (Baxter et al., 2014; Kuehner, 2017; McLean, Asnaani, Litz, & Hofmann, 2011; Wittchen et al., 2011). However, is this true over the entire lifespan? Some studies suggest that the gap between the sexes remains the same across the lifespan (Baxter et al., 2014; Cairney & Wade, 2002; Ferrari et al., 2013; Jorm, 2000; Kessler, McGonagle, Swartz, Blazer, & Nelson, 1993), but other studies found a decreased (Bebbington et al., 1998; Jorm, 2000; Wittchen & Jacobi, 2005) as well as an increased gap (Kessler et al., 2010a) in older ages.

Lastly, it is unclear whether there are significant differences in trajectories across these various highly comorbid internalizing disorders, and how these trajectories of internalizing disorders compare with the trajectories of internalizing symptoms and traits, such as depressive symptoms, anxiety symptoms, negative affect (NA), and neuroticism (Jorm, 2000; Keyes et al., 2014; Twenge et al., 2019; Wells et al., 2006). Insight in the difference between the trajectories of internalizing disorders, symptoms, and traits can inform discussions on classification, such as whether internalizing disorders and symptoms are sufficiently similar constructs so that the latter could serve as the measures of internalizing disorders for research and clinical purposes (Cai et al., 2020; Kotov et al., 2017).

The study of these questions requires large general population samples with well-measured phenotypes, and statistical methods that are able to identify potentially complex nonlinear developments. Yet to date, no studies have used advanced nonlinear statistical methods to investigate the point prevalence of different internalizing disorders, symptoms, and traits as a function of age and compared these across sex.

Our aim is to investigate the prevalence of different internalizing disorders across age and sex, and compare the results of internalizing disorders with internalizing symptoms and traits. We investigate the point prevalence of major depression (MD), dysthymia (DYS), generalized anxiety disorder (GAD), panic disorder (PD), and social phobia (SPH) diagnosed at interview by DSM-IV criteria in a sample of 146 315 participants aged 18–80 years from Lifelines, a study in the Dutch general population. We also study the rates of depressive and anxiety symptoms, NA, and neuroticism. Generalized additive models (GAMs) allow us to model nonlinear patterns and test for significant differences in the development of the different internalizing disorders, symptoms and traits, and compare results for men and women.

## Methods

### Sample

The Lifelines Cohort Study is a multidisciplinary prospective population-based cohort study of 167 729 subjects in the north of the Netherlands. It was established as a resource for research on complex interactions between environmental, phenotypic, and genomic factors in the development of chronic diseases and healthy aging. It employs a broad range of investigative procedures in assessing the biomedical, socio-demographic, behavioral, physical, and psychological factors contributing to health and disease, with a special focus on multimorbidity and complex genetics (Scholtens et al., 2015; Stolk et al., 2008). Between 2006 and 2013, an index population aged 25–49 years was recruited via participating general practitioners. Subsequently, older and younger family members were invited to participate in Lifelines. In addition, adults could self-register via the Lifelines website. In total, 49% of the included participants were invited through their GP, 38% were recruited via participating family members, and 13% self-registered (Scholtens et al., 2015). Most participants (57%) were included in 2012–2013 (Klijs et al., 2015). Baseline data were collected for 167 729 participants.

The Lifelines adult study population is broadly representative of the adult population of the north of the Netherlands. Demographic, socioeconomic, and general health characteristics of the Lifelines cohort are described elsewhere (Klijs et al., 2015). All participants provided written informed consent. The Lifelines Cohort Study was approved by the Medical Ethics Committee of the University Medical Center Groningen, The Netherlands. In the current study, we included all baseline participants aged 18–80 years (*n* = 146 315) who had available data on one or more of the internalizing disorders or symptoms. We excluded 299 participants over 80 years because of the low sample size for the statistical analyses.

### Measurements

#### Internalizing disorders

Current MD, DYS, SPH, PD, and GAD were assessed according to DSM-IV-TR criteria with a standardized diagnostic interview based on the Mini-International Neuropsychiatric Interview (MINI) (Sheehan et al., 1998). Trained medical assistants administered sections of the MINI to all participants during their visit to the research facilities and entered the responses into the computer. Conform DSM-IV-TR duration criteria, MD, DYS, GAD, and PD were rated as present if the subject reported the required symptoms in the past 2 weeks, 2 years, 6 months, and 1 month, respectively (American Psychiatric Association, 2000). SPH was assessed during the past month. We selected all 146 315 participants aged 18–80 with present data on the MINI questionnaire. For further details, see Supplementary Methods.

#### Internalizing symptoms and neuroticism

*Depressive and anxiety symptoms:* Using the symptoms of MD and GAD assessed with the MINI, we created two sum scores for depressive (range 0–9) and anxiety symptoms (range 0–7). As stated above, MD symptoms were assessed in the past 2 weeks, and GAD symptoms in the past 6 months. Due to changes in the design of the interview, only part of the sample (*n* = 73 805) had data on additional symptoms of MD and GAD if the core criteria were absent. This subsample with complete data was used for the analyses of depressive and anxiety symptoms (Supplementary Methods).

*Negative affect:* NA was assessed with the Positive and Negative Affect Schedule (PANAS) using 10 items including feeling irritable, ashamed, upset, nervous, guilty, scared, hostile, jittery, afraid, and distressed (Cronbach's *α* = 0.84–0.87) (Crawford & Henry, 2004; Watson, Clark, & Tellegen, 1988). Subjects were asked to rate how often they experienced each item in the past 4 weeks on a five-point Likert scale resulting in a score ranging from 10 to 50.

*Neuroticism:* Current neuroticism was assessed with the Revised NEO Personality Inventory (Costa & McCrae, 1992; Hoekstra, Ormel, & De Fruyt, 2007). The NEO PI-R Neuroticism subscale (Cronbach's *α* = 0.91) consists of 48 items covering the facets of anxiety, angry/hostility, depression, self-consciousness, impulsiveness, and vulnerability (Kurtz, Lee, & Sherker, 1999). Items were answered on a five-point Likert scale resulting in a sum score ranging from 48 to 240. The initial questionnaire excluded the depression and anxiety facets to limit the total length of the questionnaires, but these were added later. Here we only studied participants for whom complete data on all subscales on the NEO were available (*n* = 42 658) (see Supplementary Methods for details).

### Statistical analysis

#### Weighted point prevalence

Because women and certain age groups were overrepresented in Lifelines (see Supplementary Methods), we used a person weighting factor based on age and sex to estimate the point prevalence of internalizing disorders, symptoms, and traits for the Dutch general population. Data on the sex and age distribution of the Dutch population in 2011 were derived from the CBS Statline data [Centraal Bureau voor de Statistiek (CBS), 2020].

#### Generalized additive models

GAMs were used to assess the prevalence of internalizing disorders, symptoms, and traits as a function of age. GAMs are regression models that can identify and characterize complex nonlinear regression effects (Hastie, Tibshirani, & Friedman, 2011), by automatically determining the optimal combination of nonlinear basis functions (e.g. linear terms, polynomial terms, cubic terms, etc.) (Wieling, 2018; Wood, 2017). Overfitting is prevented by minimizing a combination of the error and a non-linearity penalty (Wieling, 2018). All analyses were performed in R_3.5.2 using the packages *mgcv_1.9.29* (Marra & Wood, 2011; Wood, 2017) and *itsadug_2.3* (van Rij, Wieling, Baayen, & van Rijn, 2016). We modeled the prevalence of each internalizing disorder, and the means of the symptom scores and neuroticism score as a (potentially) nonlinear function of age, and tested if there was a significant interaction effect between sex and age, i.e. if the patterns across age varied depending on sex. Subsequently, we modelled the patterns of the five internalizing disorders to investigate if the intercept and the pattern across age varied depending on the disorder type. For these models, the prevalence of any disorder served as the dependent variable, and the type of disorder was used as the independent variable. The reference classes were varied to make sure the results were robust.

#### Sensitivity analyses

Internalizing disorders are highly comorbid (Bijl, van Zessen, Ravelli, de Rijk, & Langendoen, 1998b; Kessler, Chiu, Demler, & Walters, 2005). Therefore, we performed a sensitivity analysis by including a random intercept for each subject in the GAM. This random intercept accounted for individual variation in vulnerability for internalizing disorders, irrespective of age, so that the fixed effect of age on internalizing disorders on a group level could be estimated. As the current software was not able to run a GAM with random effects for the full sample, we divided the sample into 10 random subsamples of 14 624 individuals each. These subsamples were matched to the full sample based on age and sex distributions. Then, we performed the GAM *without* and *with* random intercepts for these 10 subsamples, and compared the results.

Because family history is an important risk for developing internalizing disorders, we also performed a sensitivity analysis by including a random intercept in the GAMs for individual disorders in the full sample. This random intercept accounted for family variation in vulnerability for internalizing disorders.

## Results

### Point prevalence

The included 146 315 participants had a mean age of 44.2 years (s.d. 12.7) and 58.6% were women (Table 1). The age and sex weighted point prevalence rates showed that current GAD was reported most frequently (3.7%), followed by MD (2.0%), DYS (1.0%), and SPH (0.8%). PD in the past month was rare (0.21%). The point prevalence rates differed significantly between all disorders as indicated by the parametric terms for each of the disorders compared to the reference class (online Supplementary Table S2). The unweighted prevalence rates were somewhat higher for all disorders because of the sex and age composition of Lifelines participants, including a higher percentage of women than the general Dutch population (online Supplementary Table S1).

**Table 1.** Baseline characteristics

	*N*	Total	Men	Women
Demographics				
Sex	146 315		41.42%	58.58%
Age, mean (s.d.)	146 315	44.21 (12.74)	44.84 (12.78)	43.77 (12.69)
Education level, % (s.e.)^a^				
Low	142 735	29.61 (0.12)	29.68 (0.19)	29.56 (0.16)
Intermediate	142 735	40.12 (0.13)	38.67 (0.20)	41.15 (0.17)
High	142 735	30.27 (0.12)	31.65 (0.19)	29.29 (0.16)
Internalizing disorders, % (s.e.)^b^				
MD (2 weeks)	146 314	1.98 (0.04)	1.42 (0.05)	2.53 (0.06)
Dysthymia (2 years)	142 549	1.04 (0.03)	0.77 (0.04)	1.30 (0.04)
GAD (6 months)	146 315	3.71 (0.05)	2.79 (0.07)	4.62 (0.08)
PD (1 month)	146 315	0.21 (0.01)	0.15 (0.02)	0.27 (0.02)
SPH (1 month)	146 313	0.84 (0.03)	0.75 (0.04)	0.93 (0.04)
Any mood disorder	145 793	3.00 (0.05)	2.19 (0.07)	3.81 (0.08)
Any anxiety disorder	146 313	4.32 (0.06)	3.33 (0.08)	5.30 (0.08)
Any internalizing disorder	145 956	5.82 (0.07)	4.41 (0.09)	7.22 (0.10)
Internalizing traits, mean (s.d.)^b^				
MD symptoms (range: 0–9)	73 781	0.52 (1.16)	0.40 (1.01)	0.65 (1.27)
GAD symptoms (range: 0–7)	73 781	1.03 (1.75)	0.79 (1.54)	1.26 (1.91)
Neuroticism (range: 48–240)	42 658	119.65 (21.14)	115.35 (20.26)	124.15 (21.11)
Negative affect (range: 10–50)	138 859	20.54 (5.24)	19.61 (5.02)	21.47 (5.28)

DYS, dysthymia; GAD, generalized anxiety disorder; MD, major depression; PD, panic disorder; s.d., standard deviation; s.e., standard error; SPH, social phobia.

a Highest completed education: ‘Low’ is completed junior general secondary education (mavo/vmbo-t) or lower, or no education; ‘Intermediate’ is completed secondary vocational education (mbo), senior general secondary education (havo, vwo, hbs, mms); ‘High’ is completed higher vocational education (hbo) or university.

b Age and sex weighted estimates to the average Dutch population in 2011. For unweighted estimates, see online Supplementary Table S1.

### Lifetime patterns of internalizing disorders

**Fig. 1.**
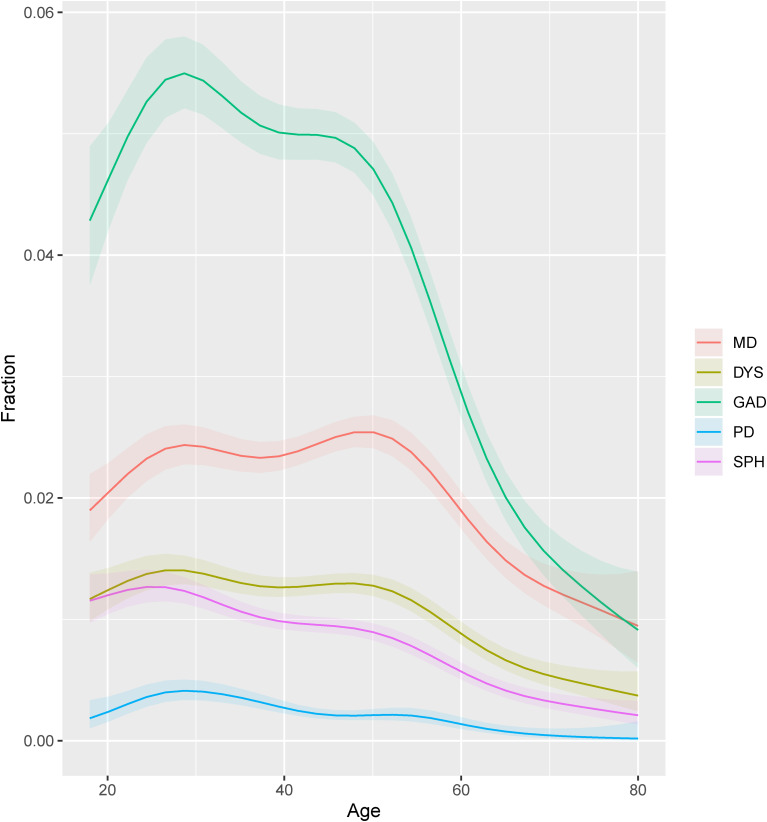
Estimated point prevalence for each internalizing disorder by age. DYS, dysthymia; GAD, generalized anxiety disorder; MD, major depression; PD, panic disorder; SPH, social phobia. Point prevalence for each internalizing disorder by age, as estimated by a generalized additive model. All patterns were nonlinear as indicated by the smoothing curves with effective degrees of freedom larger than 1 with *p* values <0.05 (online Supplementary Table S2). The smoothing curves were all significantly different from each other except for SPH-PD and for DYS-GAD.

All internalizing disorders showed significant nonlinear patterns over the lifespan (Fig. 1, online Supplementary Table S2). The general trend was that their prevalence increased from the age of 18 until the age of 30, stabilized until the age of 50, and then decreased. However, there were also differences between the disorders, as indicated by their significantly different curves. The prevalence of SPH and PD decreased relatively early in life, whereas the prevalence of MD peaked at two ages, around 30 and 50 years, a pattern not seen with other disorders. Additionally, the prevalence of GAD and DYS dropped more steeply after the age of 50 than did the other disorders. The curves for GAD-DYS and for PD-SPH were not significantly different when changing the reference class, indicating no robust difference in their curves.

### Sex differences and similarities

**Fig. 2.**
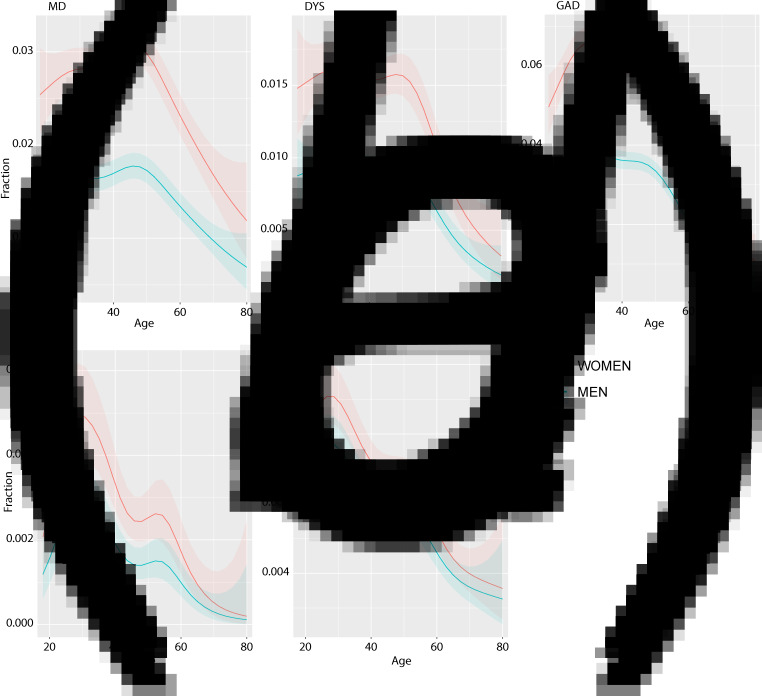
Estimated point prevalence for internalizing disorders in men and women. DYS, dysthymia; GAD, generalized anxiety disorder; MD, major depression; PD, panic disorder; SPH, social phobia. Point prevalence for both sexes for each internalizing disorder by age, as estimated by generalized additive models for each disorder separately. For all five disorders, there were differences in intercepts between men and women but smoothing curves were not significantly different (see online Supplementary Table S3). Therefore, this figure is based on the models without interaction term.

As expected, women reported more internalizing disorders than men across the entire age range. The intercepts for each disorder were all significantly different for each disorder (Fig. 2, online Supplementary Table S3). However, the curves showing the increase and decrease of prevalence over age were not significantly different between the sexes, and this was true for each internalizing disorder. This implied that the odds ratio and the relative risk (i.e. prevalence women/prevalence men) were stable across the different age groups: about 1.7 for MD, DYS, GAD, and PD, and 1.2 for SPH (online Supplementary Table S4).

### Comparison with internalizing symptoms and neuroticism

**Fig. 3.**
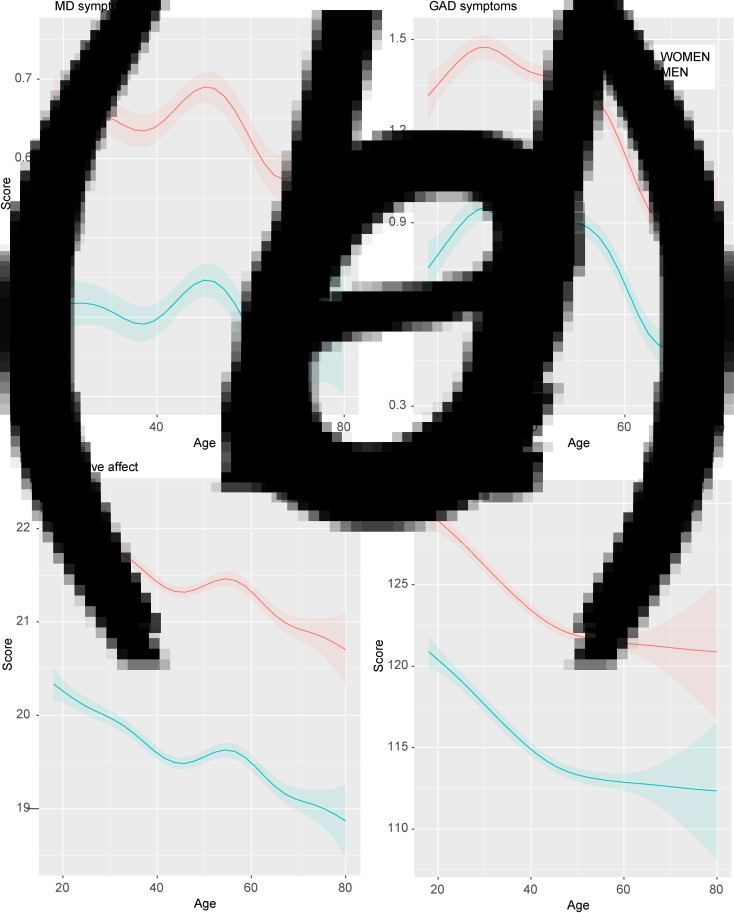
Estimated curves for internalizing symptoms and neuroticism in men and women. GAD, generalized anxiety disorder; MD, major depression. Average scores for both sexes by age, as estimated by generalized additive models for neuroticism and each symptom score separately. As can be seen in online Supplementary Table S3, there were differences in intercepts between men and women for each symptom score, as well as for neuroticism, and smoothing curves were also significantly different except for MD symptoms. Therefore, 3A is based on a model without interaction terms, while 3B-3D are based on models with interaction terms.

Internalizing symptoms and traits showed different patterns across age than did internalizing disorders (Fig. 3, online Supplementary Table S3). Depressive symptoms decreased slightly from age 18 until the age of 35, increased until the age of 50, and then decreased again until the age of 65, after which symptoms increased again. Anxiety symptoms increased until the age of 40, and then decreased, with a stabilization after age 70. Neuroticism and NA decreased largely linearly from the age of 18 years. NA diminished linearly except from an increase from the age of 45 until the age of 55, but this increase was minor (<0.5 point on a scale from 10 to 50), and neuroticism stabilized from the age of 50.

Comparable to the internalizing disorders, women scored higher on neuroticism and all internalizing symptoms than men, especially in depressive and anxiety symptoms (ratio W/M~1.6) and less for NA and neuroticism (ratio W/M~1.1)(online Supplementary Table S4). The curves for depressive symptoms were similar in men and women, meaning that the absolute difference in the number of depressive symptoms remained constant over lifetime. The curves for generalized anxiety symptoms, neuroticism, and NA were significantly different across sex, although Fig. 3 shows that these differences were modest.

### Sensitivity analyses

To investigate the potential impact of comorbidity on the different trajectories of the internalizing disorders over lifetime, we performed a sensitivity analysis comparing models excluding and including random intercepts for each subject in 10 random subsamples each including about 10% of the sample. The estimated trajectories of the prevalence of internalizing disorders over lifetime were similar in all models including and excluding random intercepts (online Supplementary Table S5). To investigate the potential impact of the family structure of the Lifelines sample on our results, we performed another sensitivity analysis comparing models excluding and including random intercept for family structure. The estimated trajectories were again similar in all models including and excluding random intercepts (online Supplementary Table S6).

## Discussion

### Main findings

In this study of 146 315 subjects from the Dutch general population aged 18–80 years, we investigated the patterns of the point prevalences of MD, DYS, GAD, SPH, and PD across different ages and sex. In general, our modeling indicated an increase in the prevalence of internalizing disorders from the age of 18 years, a plateau phase between 30 and 50 years of age, and a decrease after age 50. There were differences in the nonlinear patterns over lifetime between most disorders. Internalizing symptoms and neuroticism showed a distinctly different pattern over the lifetime compared with internalizing disorders. Although women reported more internalizing disorders and symptoms and higher neuroticism than men, the relative risk over the life course was remarkably similar.

### Comparison to previous studies

To our knowledge, no previous studies used GAM to investigate the development of different internalizing disorders and symptoms and neuroticism over lifetime and across sex. Thus, we cannot directly compare the nonlinear patterns and statistical differences between the internalizing disorders and symptoms and neuroticism with the results of previous studies. However, we can compare some key findings with previous findings.

First, our estimated point prevalences of the internalizing disorders are close to the estimates of point prevalence in previous studies. Our prevalence estimate of GAD was in the range of other studies (3.7% in Lifelines *v.* 1.7–4.1%) (de Graaf et al., 2012; McLean et al., 2011; Wittchen & Jacobi, 2005), which was also true for our prevalence estimate of DYS (1.0% in Lifelines *v.* 0.9–2.3%) (Bijl, Ravelli, & van Zessen, 1998*a*; Charlson, Ferrari, Flaxman, & Whiteford, 2013; de Graaf et al., 2012). Also the overall point prevalence of any anxiety disorder was comparable to other studies (4.3% in Lifelines *v.* 4.0–9.7%) (Baxter, Scott, Vos, & Whiteford, 2013, 2014; Bijl et al., 1998*a*). Our past month estimates of PD (0.21%) and SPH (0.84%) were lower than in a smaller Dutch study in the general population (PD 1.5%; SPH 3.7%) (Bijl et al., 1998*a*). This may be due to slightly different criteria in DSM-III-R and DSM-IV-TR for PD (American Psychiatric Association, 1987, 2000), or the use of other assessment methods. Our estimate of MD was slightly lower than in other population studies (2.0% in Lifelines *v.* 2.7–4.4%) (Bijl et al., 1998*a*; de Graaf et al., 2012; Kessler et al., 2010*a*), which may have to do with a different time frame for assessment (past 2 weeks in Lifelines *v.* past month in previous studies). The relative differences in point prevalence for men and women are also as expected (Baxter et al., 2014; Kuehner, 2003, 2017; McLean et al., 2011; Wittchen et al., 2011).

Second, similar to this study, two reviews found that the point prevalence of internalizing disorders followed a nonlinear development over lifetime following an inversed U-shape (Baxter et al., 2014; Charlson et al., 2013). Anxiety disorders manifested an initial rise in prevalence until age 30, followed by a decrease which was more pronounced after age 50, similar to our findings (Baxter et al., 2014). The pattern for MD was slightly different – a rise in the prevalence of MD until age 50, followed by a decrease, and a second rise after age 75. This review also suggested similar curves for men and women across the lifespan (Baxter et al., 2014). Another review described an increase in the prevalence of DYS at early ages with a peak around 50 years (Charlson et al., 2013). Unlike our study, these reviews included studies with substantial heterogeneity, used relatively few data points [e.g. 141 (Charlson et al., 2013)], and did not formally test for complex nonlinearity or sex differences in their results.

### Implication of findings

Since this is the first study that used advanced nonlinear models to investigate the prevalence of internalizing disorders, symptoms, and traits across age and sex, we should be careful in drawing definitive conclusions. But if the results prove to be robust, they may have several implications.

First, the fact that the relative gap between the sexes remains stable over the lifetime has implications for hypotheses about risk factors for internalizing disorders. Women clearly report more internalizing disorders than men. Previous studies showed that the gap in MD prevalence between the sexes arises in puberty, due to higher incidence rates in women (Altemus, Sarvaiya, & Neill Epperson, 2014; Kessler, 2003; Kuehner, 2017). One of the hypotheses for this gap between the sexes are changes in female sex hormones during lifetime, for instance around the menopause. There are suggestions that estrogens are neuroprotective, and a decrease in estrogens in menopause would increase women's risk of MD (Georgakis et al., 2016). Several cross-sectional and longitudinal studies have studied the prevalence of MD and anxiety disorders around the menopause in women, but results are inconsistent (Bryant et al., 2012; Judd et al., 2012; Kuehner, 2017; Rössler et al., 2016; Vivian-Taylor & Hickey, 2014). Our study shows that around the age of the menopause, women indeed report more MD and depressive symptoms. However, there is a similar rise in MD and depressive symptoms in men in this age group. This implies that perimenopausal changes in female sex hormones probably do not significantly influence women's risk of depression, unless male hormonal changes or other male-specific risk factors exist that explain the similar increase in depression prevalence in middle-aged men. It is more likely that shared risk factors – e.g. psychosocial distress (Rössler et al., 2016) – explain the similar rise in depression prevalence in both sexes during midlife. A similar argument can be made for anxiety disorders, in which the relative gap between the sexes is also stable across age.

Second, the prevalence of most internalizing disorders showed different patterns over lifetime, which suggests that these disorders are not entirely identical constructs, but may have meaningful differences in etiology. At the same time, the similarity of the general pattern among the internalizing disorders suggests that there are likely shared risk factors (Kendler et al., 2011; Schoevers, Beekman, Deeg, Jonker, & Van Tilburg, 2003; Vink, Aartsen, & Schoevers, 2008).

Third, the lifetime patterns of internalizing disorders differed from those of the internalizing symptoms and neuroticism, suggesting that the relationship between these is complex, or at least not stable across the lifespan. For instance, the prevalence of depressive symptoms, but not MD, was rising after the age of 65. This may be due to the fact that older subjects report somatic symptoms of depression more often without having episodes of MD (Balsis & Cully, 2008; Hegeman, Kok, Van Der Mast, & Giltay, 2012). In any case, the fact that internalizing disorders show different patterns across age and sex than internalizing symptoms and neuroticism is relevant for the debate on the nature and classification of internalizing disorders. In this debate, psychopathology is assumed to exist on a continuum instead of there being clear boundaries between health and disease (Kendell & Jablensky, 2003; Kotov et al., 2017). Although we only investigated differences in prevalence rates, our data show that there may be important differences between internalizing disorders and symptoms and traits. This difference implies that we should be cautious in reducing internalizing disorders to high scores on symptom dimensions (Kotov et al., 2017; Schoevers et al., 2003). This concern is supported by genetic studies showing that depressive symptoms are not always good proxies for MD (Cai et al., 2020; Kendler et al., 2019).

### Strengths and limitations

This is the first study that used advanced nonlinear models to investigate the development of internalizing disorders and symptoms and neuroticism over lifetime in a large sample from the general population. The disorders were assessed with structured interviews by trained research assistants, and focused on current psychopathology to minimize recall bias (Kessler, Petukhova, Sampson, Zaslavsky, & Wittchen, 2012; Kruijshaar et al., 2005).

We also note a number of limitations. Our study uses cross-sectional data, and therefore cannot exclude period or cohort effects as an explanation for the change in point prevalence estimates across different ages. It is unlikely, however, that our findings are exclusively based on period and cohort effects. A recent study in 611 880 subjects from the US population controlling for period and cohort effects showed that the prevalence of depressive episodes followed an inverse U-shaped curve with increasing prevalence from the age of 18 and decreased after age 32, and that psychological distress declined with age (Twenge et al., 2019). Also population studies that were performed two decades apart indicate that the reduction of internalizing disorders is associated with older age (Baxter et al., 2014; Bijl et al., 1998a; de Graaf et al., 2012). Future assessment waves of Lifelines would allow an investigation of age, period, and cohort effects.

Similar to these population studies, we observed a reduction in the prevalence of internalizing disorders at older age. There are two types of explanations for the decline of internalizing disorders; (1) age is protective against internalizing disorders, (2) age is not protective, but internalizing disorders are less frequently measured in older participants due to biases. Selection bias occurs when older individuals with MD are relatively less often participating in population studies than younger individuals with MD, for example, when there is increased difficulty in establishing contact or increased refusals (Beekman et al., 2002; Holwerda et al., 2007; Schoevers et al., 2000). Reporting bias might be a result of older people being less likely to report symptoms of depression (Knäuper & Wittchen, 1994; Lyness et al., 1995). It is also possible that the prevalence of depression at older age is lower because individuals suffering from depression are more likely to have died earlier due to related causes such as heart problems (i.e. survivor bias) (White, Schulz, Klein, & von Klitzing, 2019; Wray et al., 2018). However, in Lifelines, we found no interaction effect between age and the presence of an internalizing disorder at baseline when predicting participation at follow up (2014–2017) (data not shown). This means that the impact of having a disorder on attrition for any reason was not different for older as compared to younger subjects, which makes selection bias a less likely explanation for the reduction in prevalence after age 50. Follow-up studies are needed to investigate explanations for the decline of internalizing disorders, symptoms, and traits in older participants.

Third, we assessed current internalizing disorders based on structured interviews with trained research assistants, which can be considered a strength. However, there were two limitations in the assessments. Disability was not assessed for MD and GAD, and DYS was not assessed in subjects who satisfied the criteria for MD, which could have biased prevalence rates upwards and downwards, respectively. It is most likely that these biases have been minor given that our estimates of MD, GAD, and DYS are comparable to previous estimates (Baxter et al., 2014; Bijl et al., 1998a; Charlson et al., 2013; de Graaf et al., 2012).

Fourth, the presence of internalizing symptoms may influence subjects' reports on internalizing traits like neuroticism, which could complicate disentangling between states and traits. Previous studies showed that subjects with depressive symptoms may temporarily score higher on neuroticism (Jeronimus, Ormel, Aleman, Penninx, & Riese, 2013; Kotov, Gamez, Schmidt, & Watson, 2010). If internalizing symptoms indeed have a strong effect on neuroticism, then we would have expected to see a similarity between the patterns of internalizing symptoms and neuroticism across age. However, in our study, neuroticism scores were not showing the same patterns as depressive symptoms, generalized anxiety symptoms, or NA. For example, neuroticism scores were lower in subjects aged 30–50 years than in younger subjects, whereas depressive symptoms were higher in this age group. Although these findings do not fully exclude that internalizing symptoms may have influenced neuroticism scores, it shows that the influence in our study is probably modest.

## Conclusion

This study identified different patterns in point prevalence for most internalizing disorders, symptoms, and traits over lifetime. The overall prevalence of internalizing disorders, symptoms, and traits in women was higher than in men, but the patterns across age were remarkably similar in both sexes. These results indicate that certain hypotheses for the gap between the sexes, e.g. the changes in female sex hormones during menopause, are unlikely explanations. Future studies are needed to investigate the causes for the initial rise in internalizing disorders and their decline at older age, taking into account the sex similarities.

## Supplementary material

For supplementary material accompanying this paper visit https://doi.org/10.1017/S0033291721001148. click here to view supplementary material
