# Prevalence of internalizing disorders, symptoms, and traits across age using advanced nonlinear models – ERRATUM

**DOI:** 10.1017/S003329172610350X

**Published:** 2026-04-10

**Authors:** Hanna M. van Loo, Lian Beijers, Martijn Wieling, Trynke R. de Jong, Robert A. Schoevers, Kenneth S. Kendler

**Affiliations:** 1 Department of Psychiatry, University of Groningen, University Medical Center Groningen, Groningen, The Netherlands; 2 Department of Information Science, University of Groningen, Groningen, The Netherlands; 3 Lifelines Cohort & Biobank, Roden, The Netherlands; 4 University of Groningen, University Medical Center Groningen, Research School of Behavioural and Cognitive Neurosciences (BCN), Groningen, The Netherlands; 5 Virginia Institute for Psychiatric and Behavioral Genetics, Virginia Commonwealth University, Richmond, VA, USA

The authors of the Psychological Medicine article “Prevalence of internalizing disorders, symptoms, and traits across age using advanced nonlinear models” (Van Loo et al., 2023) have identified an error in one of the analyses and associated results reported. The error specifically related to the prevalence of panic disorder. The original manuscript reports that the prevalence of panic disorder was 0.21%. An error was found in the code to derive the diagnosis of panic disorder through which subjects with a relatively low number of additional symptoms were coded as controls instead of cases. Correcting this error resulted in a higher point prevalence of panic disorder (now 0.60%), and also resulted in slightly higher prevalence rates of any anxiety disorder and any internalizing disorder (updated Table 1). The curve representing the prevalence rate of panic disorder across age was largely similar, except that the curves of PD and SPH were now subtly different from each other in terms of shape (updated Supplemental Table 2, Figure 1). Furthermore, we found a subtle difference between the curves of men and women for panic disorder (updated Supplemental Table 3 and Figure 2). However, this difference was borderline significant (*P* = 0.04), and it does not alter our overall interpretation of the results. The correction does not affect any of the other results reported in this study.

**Table 1.** Baseline characteristics

	*N* total	Total	Men	Women
Demographics				
Sex	146315		41.42%	58.58%
Age, mean (SD)	146315	44.21 (12.74)	44.84 (12.78)	43.77 (12.69)
Education level, % (SE)^a^				
Low	142735	29.61 (0.12)	29.68 (0.19)	29.56 (0.16)
Intermediate	142735	40.12 (0.13)	38.67 (0.20)	41.15 (0.17)
High	142735	30.27 (0.12)	31.65 (0.19)	29.29 (0.16)
Internalizing disorders, % (SE)^b^				
MD (2 week)	146314	1.98 (0.04)	1.42 (0.05)	2.53 (0.06)
Dysthymia (2 year)	142549	1.04 (0.03)	0.77 (0.04)	1.30 (0.04)
GAD (6 month)	146315	3.71 (0.05)	2.79 (0.07)	4.62 (0.08)
PD (1 month)	146315	0.60 (0.02)	0.44 (0.03)	0.75 (0.03)
SPH (1 month)	146313	0.84 (0.03)	0.75 (0.04)	0.93 (0.04)
Any mood disorder	145793	3.00 (0.05)	2.19 (0.07)	3.81 (0.08)
Any anxiety disorder	146313	4.56 (0.06)	3.50 (0.08)	5.60 (0.08)
Any internalizing disorder	145956	6.03 (0.07)	4.56 (0.09)	7.48 (0.10)
Internalizing traits, mean (SD)^b^				
MD symptoms (range: 0–9)	73805	0.52 (1.16)	0.4 (1.01)	0.65 (1.27)
GAD symptoms (range: 0–7)	73805	1.03 (1.75)	0.79 (1.54)	1.26 (1.91)
Neuroticism (range: 48–240)	42658	119.65 (21.14)	115.35 (20.26)	124.15 (21.11)
Negative Affect (range: 10–50)	138859	20.54 (5.24)	19.61 (5.02)	21.47 (5.28)

DYS, dysthymia; GAD, generalized anxiety disorder; MD, major depression; NA, negative affect; PD, panic disorder; SD, standard deviation; SE, standard error; SPH, social phobia.

^a^ Highest completed education: Low = junior general secondary education (mavo/vmbo-t) or lower, or no education; Intermediate = secondary vocational education (mbo), senior general secondary education (havo, vwo, hbs, mms); High = higher vocational education (hbo) or university. The estimates in this table are unweighted for age and sex as opposed to the estimates in Table 1.

^b^ Age and sex weighted estimates to the average Dutch population in 2011. For unweighted estimates, see online Supplementary Table S1.

**Figure 1.**
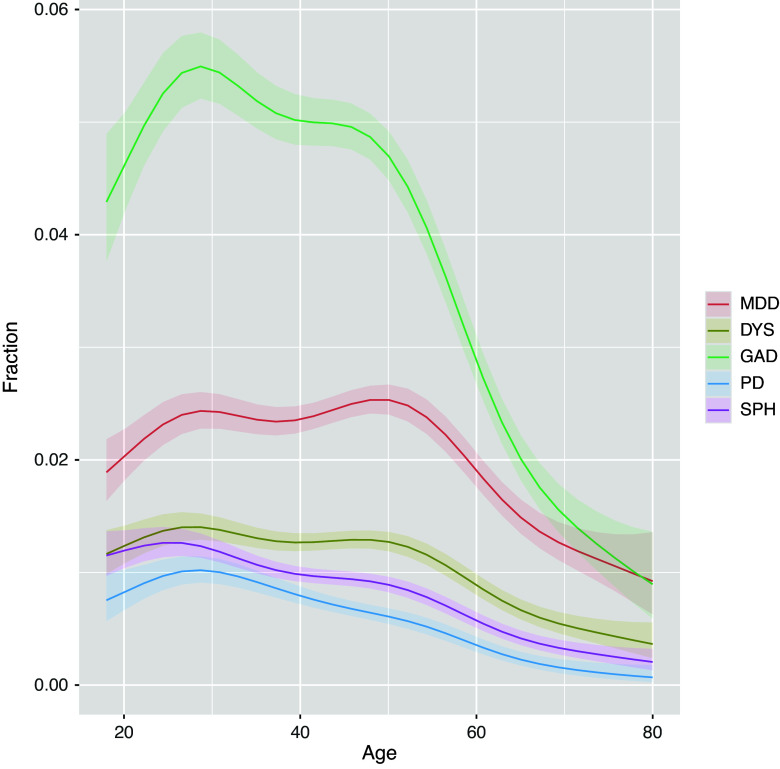
Estimated point prevalence for each internalizing disorder by age. DYS, dysthymia; GAD, generalized anxiety disorder; MD, major depression; PD, panic disorder; SPH, social phobia. Point prevalence for each internalizing disorder by age, as estimated by a generalized additive model. All patterns were nonlinear as indicated by the smoothing curves with effective degrees of freedom larger than 1 with *p* values <0.05 (online Supplementary Table S2). The smoothing curves were all significantly different from each other except for DYS-GAD.

**Figure 2.**
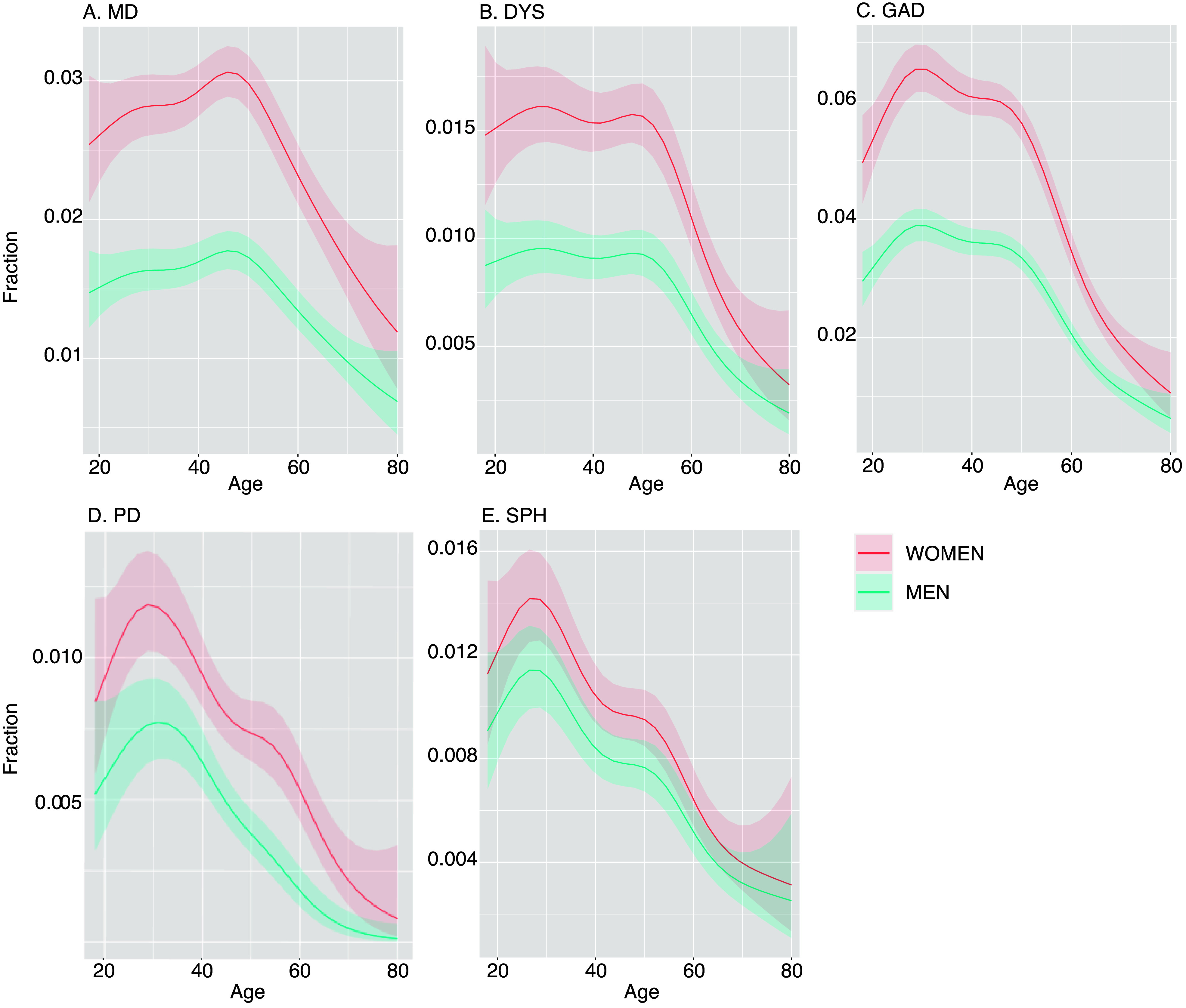
Estimated point prevalence for internalizing disorders in men and women. DYS, dysthymia; GAD, generalized anxiety disorder; MD, major depression; PD, panic disorder; SPH, social phobia. Point prevalence for both sexes for each internalizing disorder by age, as estimated by generalized additive models for each disorder separately. For all five disorders, there were differences in intercepts between men and women but smoothing curves were not significantly different, except for a borderline significant difference in PD (*P* = 0.04)(see Supplementary Table S3). Therefore, this figure is based on the models without interaction term, except for the figure for panic disorder (2D).

## References

[r1] Van Loo, H. M., Beijers, L., Wieling, M., De Jong, T. R., Schoevers, R. A., & Kendler, K. S. (2023). Prevalence of internalizing disorders, symptoms, and traits across age using advanced nonlinear models. Psychological Medicine, 53(1), 78–87. 10.1017/S0033291721001148 33849670 PMC9874996

