# Supplementary material for: Prevalence of internalizing disorders, symptoms, and traits across age using advanced nonlinear models
Source: Psychol Med. 2021 Apr 14;53(1):78–87. doi: 10.1017/S0033291721001148 (PMC9874996; doi:10.1017/S0033291721001148)
Supplement: Supplementary file 1 [file S0033291721001148sup.zip › S0033291721001148sup001.pdf]

SUPPLEMENTAL METHODS

This Supplemental Methods file includes additional details on the measurements and statistical methods that are described in the Methods section of the manuscript.

Measurements

Internalizing disorders

Current major depression (MD), dysthymia (DYS), social phobia (SPH), and generalized anxiety disorder (GAD) were assessed according to DSM-IV-TR criteria with a standardized diagnostic interview based on the Mini-International Neuropsychiatric Interview (MINI)(Sheehan *et al.* 1998). Trained research assistants administered sections of the MINI to all participants during their visit to the research facilities, and entered the responses into the computer. Conform DSM-IV-TR duration criteria, MD, DYS, and GAD, PD, were rated as present if the subject reported the required symptoms in the past 2 weeks, 2 years, 6 months, and 1 month, respectively (American Psychiatric Association 2000). SPH was assessed during the past month.

We included disability criteria for DYS and SPH. We did not take into account the criterion of disability or interference for MD, GAD, and PD, as these items were not assessed (note that the DSM-IV-TR does not include a disability criterion for PD). In addition, dysthymia was not assessed in subjects who satisfied criteria for MD in the past two weeks, because of the difficulty in determining whether the mood disturbance is accounted for by chronic MD, or by dysthymia. Criterion D for DYS in the DSM states that "no MD episode has been present during the first two years of the disturbance; i.e. the disturbance is not better accounted for by chronic MD, or MD in partial remission")(American Psychiatric Association 2000). DYS was not assessed if a diagnosis of MDD hasd been established (MINI version 3) or at least 1 core criterion and 3 additional criteria of MDD were present (MINI version 2) (total *n*=3748). We did not use exclusion criteria for alcohol/somatic disorders.

Internalizing traits

Negative affect

The Positive and Negative Affect Schedule (PANAS)(Watson *et al.* 1988; Crawford & Henry 2004) was a self-report instrument (paper questionnaire) and participants completed this questionnaire at home. Subjects were asked to rate how often they experienced each item in the past 4 weeks on a 5-point Likert scale (never, rarely, sometimes, often, very often) resulting in a score ranging from 10-50.

Neuroticism

Current neuroticism was assessed with the Revised NEO Personality Inventory (Costa & McCrae 1992; Hoekstra *et al.* 2007). Items were answered on a 5-point Likert scale that ranged from strongly disagree

to strongly agree, resulting in a sum score ranging from 48 to 240. The initial questionnaire excluded the depression and anxiety facets to limit the total length of the questionnaires for participants, but were later added. Here we only studied participants for whom complete data on all subscales on the NEO were available (n=42,658). We excluded 87,248 subjects because of missing information on the anxiety and depression subscales; this missingness was due to the design of the initial questionnaire. The initial questionnaire did not include items about depression and anxiety subscales to reduce the burden for participants. Later it was decided to include all subscales. Another 3511 subjects were excluded because they had missing data on one of the other subscales of the NEO.

Missing data

Because of the design of the questionnaires, not all participants had data on each internalizing trait or disorder (see Table 1 for sample sizes of each analysis). Table 1 shows the number of missing data points due to different reasons. Because of our primary interest in internalizing disorders, we only included subjects who had data on the MINI. There are some subjects with a number of missing values on the MINI, because of the skip structure of the questionnaire. In one version of the MINI (n = 72,510), the additional symptoms of MD and GAD were only asked if at least one core criterion of MD or GAD was present. This did not interfere with establishing of a diagnosis according to DSM-IV-TR criteria. Furthermore, DYS was not assessed if a diagnosis of MDD present (n=3,748) (see above). This also had consequences for the any depression and any internalizing psychopathology variables. A number of the subjects who had data on the MINI did not answer the questionnaires about education level (n=483), neuroticism (n=15,222), or negative affect (n=5,052). Another 84,924 subjects answered a questionnaire about neuroticism that did not include the anxiety and depression subscales (see above). Finally, we coded the value 'other' for education level as missing (n=2,912). We decided not to impute data for any of our variables, because the number of true missings was low.

Supplemental Table 1. Missing data

	Present Total	Missing Total	Missing Not answered	Missing By design	Missing True
**Sex**	146315	0	0	0	0
**Age**	146315	0	0	0	0
**Education**	142735	3580	483	2912	185
**MD**	146314	1	0	0	1
**Dysthymia**	142549	3766	0	3748	18
**GAD**	146315	0	0	0	0
**PD**	146315	0	0	0	0
**SPH**	146313	2	0	0	2
**Any mood disorder**	145793	522	0	503	19

**Any anxiety disorder**	146313	2	0	0	2
**Any internalizing disorder**	145956	359	0	348	11
**MD symptoms**	73805	72534	0	72510	24
**GAD symptoms**	73805	72536	0	72510	26
**Neuroticism**	42658	103657	15222	84924	3511
**Negative affect**	138859	7456	5052	0	2404

Sex

Recent studies show that 'sex' and 'gender' are differently related to health outcomes (Ballering *et al.* 2020), although these two concepts are deeply intertwined (Kuehner 2017). Sex refers to biological differences between men and women, whereas gender refers to psychosocial and cultural differences in roles, identities, and behaviors between men and women.

The current study focuses on biological sex. In Lifelines, participants were asked about their sex with the question "What is your sex?" with two answer options "male" or "female". The Dutch question in Lifelines was: "Wat is uw geslacht?" with two answer options "man" or "vrouw". There was no option for participants to indicate intersex variations, which occur in about 1.7% of the general population (Ballering *et al.* 2020).

Statistical analysis

Point prevalence

Because women and certain age groups were overrepresented in Lifelines (see Supplemental Figure 1), we used a person weighting factor based on age and sex in order to estimate the point prevalence of internalizing disorders and traits for the Dutch general population. Weighting was only done for the entire sample at once, rather than for each variable separately, because the age and sex distributions in the groups with and without missing data were similar. These analyses were performed in R_3.5.2 using survey_3.37.(Lumley 2004) By default, the survey package employs the Taylor series linearization method. Data on the sex and age distribution of the Dutch population were derived from the Statistics Netherlands (*Centraal Bureau voor de Statistiek*) data from the year 2011 (Centraal Bureau voor de Statistiek (CBS) 2020). A matrix was constructed with 126 strata (age 18-80, gender). For each stratum (e.g. 25-year-old males) a weighting factor was calculated using the formula:

$$w_{ag} = \left(\frac{N_{ag}}{n_{ag}}\right) * n/N$$

where w_{ag} is the weighting factor for persons in Lifelines with age a and gender g. N_{ag} and n_{ag} are the number of persons in the Netherlands and Lifelines with age a and gender g, respectively. N and n are the total population in the Netherlands in 2011, and the total number of respondents in Lifelines.

Supplemental Figure 1. Age distribution of the sample

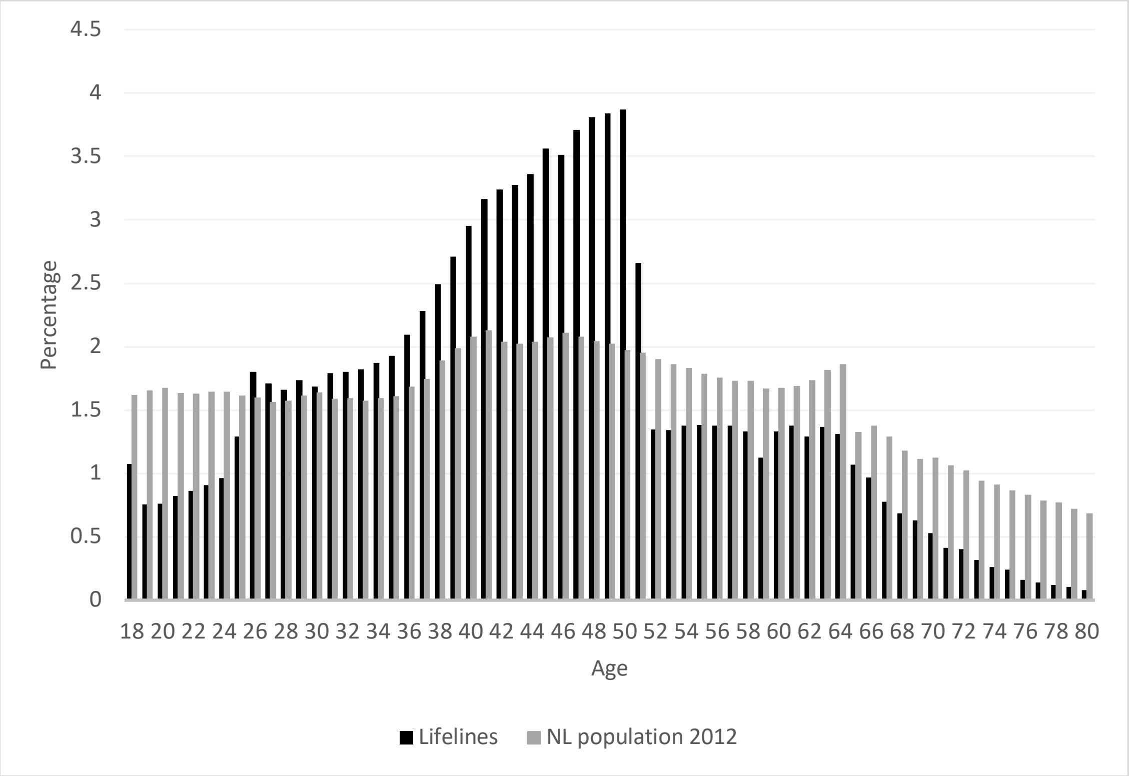

This figure represents the age distribution of all 146,315 participants aged 18-80 included in this study. Between 2006 and 2013, an index population aged 25–49 years was recruited via participating general practitioners. Subsequently, older and younger family members were invited to participate in Lifelines. In addition, adults could self-register via the Lifelines website. In total, 49% of the included participants were invited through their GP, 38% were recruited via participating family members, and 13% self-registered. Most participants (57%) were included in 2012-2013 (Klijs et al., 2005). Baseline data were collected for 167,729 participants (91.2% adults; age range 6 months–93 years). Data on the age distribution of the Dutch population in 2011 were derived from the CBS Statline data (Centraal Bureau voor de Statistiek (CBS) 2020).

Generalized additive models

To investigate the point prevalence of individual internalizing disorders across age, we modeled the point prevalence (dependent variable) of these disorders as a nonlinear function of age and sex (independent variables), using generalized additive models (GAM). For the GAM models weighting was not needed, because the adjustment for age and sex is already included in the model. All analyses were performed in R using *mgcv* version 1.9.29 (Marra & Wood 2011; Wood 2017) and *itsadug* version 2.3 (van Rij *et al.* 2016).

First, we investigated the lifetime pattern for each internalizing disorder in additive logistic regression models, and investigated whether there were sex differences by allowing separate intercepts and smoothing curves for men and women across age, using the following formula:

```
Model1 <- bam(MDD ~ SEX + s(AGE) + s(AGE,by=SEX), data=data, family='binomial')
```

We performed similar analyses for internalizing traits (i.e. MD and GAD symptom counts, neuroticism and positive affect), but used additive linear regression models as these traits were continuous instead of binary, using the following formula:

```
Model2 <- bam(Negative_affect ~ s(AGE) + s(AGE,by=SEX) + SEX, data=mydata,
family='gaussian')
```

Subsequently, we compared the trajectories of the five internalizing disorders over lifetime. We created a factor variable indicating the presence of all internalizing disorders for each subject, and allowed different intercepts and curves for each disorder over lifetime. We also checked whether the differences between internalizing disorders depended on the reference class of the model. We used the formula below in which TYPE represents the factor variable of the 5 internalizing disorders. We ran this model 5 times, with changing reference classes for TYPE.

```
Model3 <- bam(DISORDER ~ s(AGE) + s(AGE,by=TYPE) + TYPE, data=mydata_long,
family='binomial')
```

The advantage of GAM over fitting more simple nonlinear models is that GAM can combine several nonlinear patterns simultaneously, instead of specifying one type of nonlinear pattern a priori. However, because of this flexibility, GAM includes a penalty on nonlinearity to prevent overfitting (i.e., to prevent that the model picks up on idiosyncrasies of the data).

References

American Psychiatric Association (2000). *Diagnostic and Statistical Manual of Mental Disorders (DSM-IV-TR)*. 4th, text edn. American Psychiatric Association: Washington, D.C.

Ballering A V., Bonvanie IJ, Olde Hartman TC, Monden R, Rosmalen JGM (2020). Gender and sex independently associate with common somatic symptoms and lifetime prevalence of chronic disease. Elsevier Ltd *Social Science and Medicine* **253**, 112968.

Centraal Bureau voor de Statistiek (CBS) (2020). *StatLine - Nederlandse bevolking 2011 naar geslacht en leeftijd*. Statline

Costa PT, McCrae RR (1992). *Revised NEO personality inventory (NEO-PI-R) and NEO five-factor inventory (NEO-FFI). Odessa FL Psychological Assessment Resources*

Crawford JR, Henry JD (2004). The Positive and Negative Affect Schedule (PANAS): Construct validity, measurement properties and normative data in a large non-clinical sample. *British Journal of Clinical Psychology* **43**, 245–265.

Hoekstra H, Ormel J, Fruyt F De (2007). NEO-PI-R / NEO-FFI: Big Five Personality Inventory Manual. *Lisse: Swets & Zeitlinger*

Kuehner C (2017). Why is depression more commen among women than among men? Elsevier Ltd *The Lancet Psychiatry* **4**, 146–58.

Lumley T (2004). Analysis of complex survey samples. *Journal of Statistical Software* **9**, 1–19.

Marra G, Wood SN (2011). Practical variable selection for generalized additive models. *Computational Statistics and Data Analysis* **55**, 2372–2387.

van Rij J, Wieling M, Baayen RH, van Rijn H (2016). *itsadug: Interpreting time series and autocorrelated data using GAMMS. R package* 2.3

Sheehan D V, Lecrubier Y, Sheehan KH, Amorim P, Janavs J, Weiller E, Hergueta T, Baker R, Dunbar GC (1998). The Mini-International Neuropsychiatric Interview (M.I.N.I.): the development and validation of a structured diagnostic psychiatric interview for DSM-IV and ICD-10. . University of South Florida College of Medicine, Tampa 33613, USA. *The Journal of clinical psychiatry* **59 Suppl 2**, 22–57.

Watson D, Clark LA, Tellegen A (1988). Development and validation of brief measures of positive and negative affect: The PANAS scales. *Journal of Personality and Social Psychology* **47**, 1063–1070.

Wood SN (2017). *Generalized additive models: An introduction with R, second edition*. CRC Press.
