# Supplementary material for: Prevalence of internalizing disorders, symptoms, and traits across age using advanced nonlinear models
Source: Psychol Med. 2021 Apr 14;53(1):78–87. doi: 10.1017/S0033291721001148 (PMC9874996; doi:10.1017/S0033291721001148)
Supplement: Supplementary file 1 [file S0033291721001148sup.zip › S0033291721001148sup002.docx]

**SUPPLEMENTAL TABLE 1. Baseline characteristics (unweighted)**

|  | **N total** | **Total** | **Men** | **Women** |
| --- | --- | --- | --- | --- |
| **Demographics** |  |  |  |  |
| Sex | 146315 |  | 41.42% | 58.58% |
| Age, mean (sd) | 146315 | 44.21 (12.74) | 44.84 (12.78) | 43.77 (12.69) |
| Education level, % (se)* |  |  |  |  |
| Low | 142735 | 29.61 (0.12) | 29.68 (0.19) | 29.56 (0.16) |
| Intermediate | 142735 | 40.12 (0.13) | 38.67 (0.20) | 41.15 (0.17) |
| High | 142735 | 30.27 (0.12) | 31.65 (0.19) | 29.29 (0.16) |
| **Internalizing disorders, % (se)**  MD (2 week) | 146314 | 2.22 (0.04) | 1.56 (0.05) | 2.68 (0.06) |
| Dysthymia (2 year) | 142549 | 1.18 (0.03) | 0.84 (0.04) | 1.42 (0.04) |
| GAD (6 month) | 146315 | 4.29 (0.05) | 3.08 (0.07) | 5.14 (0.08) |
| PD (1 month) | 146315 | 0.24 (0.01) | 0.17 (0.02) | 0.30 (0.02) |
| SPH (1 month) | 146313 | 0.91 (0.02) | 0.79 (0.04) | 0.99 (0.03) |
| Any mood disorder | 145793 | 3.38 (0.05) | 2.39 (0.06) | 4.08 (0.07) |
| Any anxiety disorder | 146313 | 4.93 (0.06) | 3.64 (0.08) | 5.85 (0.08) |
| Any internalizing disorder | 145956 | 6.57 (0.06) | 4.79 (0.09) | 7.83 (0.09) |
| **Internalizing traits, mean (sd)***  MD symptoms (range: 0-9) | 73805 | 0.55 (1.20) | 0.41 (1.03) | 0.65 (1.30) |
| GAD symptoms (range: 0-7) | 73805 | 1.12 (1.84) | 0.84 (1.59) | 1.33 (1.98) |
| Neuroticism (range: 48-240) | 42658 | 120.07 (21.32) | 114.98 (20.33) | 123.71 (21.26) |
| Negative Affect (range: 10-50) | 138859 | 20.71 (5.31) | 19.62 (5.05) | 21.48 (5.35) |

*DYS, dysthymia; GAD, generalized anxiety disorder; MD, major depression; NA, negative affect; PD, panic disorder; sd, standard deviation; se, standard error; SPH, social phobia.*

**Highest completed education: Low = junior general secondary education (mavo/vmbo-t) or lower, or no education; Intermediate = secondary vocational education (mbo), senior general secondary education (havo, vwo, hbs, mms); High = higher vocational education (hbo) or university. The estimates in this table are unweighted for age and sex as opposed to the estimates in Table 1.*

**SUPPLEMENTAL TABLE 2. Generalized additive models testing differences between internalizing disorders**

| **MD** | **Estimate** | **SE** | **z** | **P** |  |
| --- | --- | --- | --- | --- | --- |
| (Intercept) | -3.80 | 0.02 | -211.74 | <2e-16 |  |
| DYS | -0.66 | 0.03 | -21.33 | <2e-16 |  |
| GAD | 0.65 | 0.02 | 28.87 | <2e-16 |  |
| PD | -2.34 | 0.06 | -36.42 | <2e-16 |  |
| SPH | -0.95 | 0.03 | -27.87 | <2e-16 |  |
| **Smooth terms** | **edf** | **Ref.df** | **Chi.sq** | **P** |  |
| s(AGE) | 6.40 | 7.43 | 132.79 | <2e-16 |  |
| s(AGE): DYS | 1.26 | 1.47 | 6.01 | 0.02 |  |
| s(AGE): GAD | 2.75 | 3.46 | 47.90 | 0.00 |  |
| s(AGE): PD | 4.62 | 5.64 | 36.77 | 0.00 |  |
| s(AGE): SPH | 1.23 | 1.42 | 36.38 | 0.00 |  |
|  |  |  |  |  |  |
| **Models with different reference classes** | | |  |  |  |
| **DYS** | **Estimate** | **SE** | **z** | **P** |  |
| (Intercept) | -4.45 | 0.03 | -178.03 | <2e-16 |  |
| MD | 0.65 | 0.03 | 21.35 | <2e-16 |  |
| GAD | 1.30 | 0.03 | 45.83 | <2e-16 |  |
| PD | -1.68 | 0.07 | -25.31 | <2e-16 |  |
| SPH | -0.29 | 0.04 | -7.61 | <2e-16 |  |
| **Smooth terms** | **edf** | **Ref.df** | **Chi.sq** | **P** |  |
| s(AGE) | 6.41 | 7.44 | 154.18 | <2e-16 |  |
| s(AGE): MD | 1.02 | 1.04 | 6.66 | 0.01 |  |
| s(AGE): GAD | 2.71 | 3.41 | 13.78 | 0.01 |  |
| s(AGE): PD | 4.62 | 5.64 | 24.05 | 0.00 |  |
| s(AGE): SPH | 1.35 | 1.61 | 11.19 | 0.01 |  |
| **GAD** | **Estimate** | **SE** | **z** | **P** |  |
| (Intercept) | -3.15 | 0.01 | -232.53 | <2e-16 |  |
| MD | -0.65 | 0.02 | -28.88 | <2e-16 |  |
| DYS | -1.31 | 0.03 | -45.98 | <2e-16 |  |
| PD | -2.99 | 0.06 | -47.34 | <2e-16 |  |
| SPH | -1.59 | 0.03 | -49.65 | <2e-16 |  |
| **Smooth terms** | **edf** | **Ref.df** | **Chi.sq** | **P** |  |
| s(AGE) | 6.54 | 7.55 | 390.91 | <2e-16 |  |
| s(AGE): MD | 2.76 | 3.48 | 45.28 | 0.00 |  |
| s(AGE): DYS | 1.65 | 2.07 | 4.34 | *0.11* | *NS* |
| s(AGE): PD | 4.58 | 5.60 | 19.38 | 0.00 |  |
| s(AGE): SPH | 2.87 | 3.61 | 10.85 | 0.02 |  |
| **PD** | **Estimate** | **SE** | **z** | **P** |  |
| (Intercept) | -6.12 | 0.06 | -106.27 | <2e-16 |  |
| MD | 2.32 | 0.06 | 38.43 | <2e-16 |  |
| DYS | 1.66 | 0.06 | 26.48 | <2e-16 |  |
| PD | 2.96 | 0.06 | 50.18 | <2e-16 |  |
| SPH | 1.37 | 0.06 | 21.29 | <2e-16 |  |
| **Smooth terms** | **edf** | **Ref.df** | **Chi.sq** | **P** |  |
| s(AGE) | 6.53 | 7.54 | 145.45 | <2e-16 |  |
| s(AGE): MD | 1.93 | 2.42 | 27.81 | 0.00 |  |
| s(AGE): DYS | 1.03 | 1.04 | 13.56 | 0.00 |  |
| s(AGE): GAD | 2.22 | 2.79 | 12.87 | 0.00 |  |
| s(AGE): SPH | 2.10 | 2.64 | 4.99 | *0.17* | *NS* |
|  |  |  |  |  |  |
|  |  |  |  |  |  |
| **SPH** | **Estimate** | **SE** | **z** | **P** |  |
| (Intercept) | -4.75 | 0.03 | -164.45 | <2e-16 |  |
| MD | 0.95 | 0.03 | 27.97 | <2e-16 |  |
| DYS | 0.29 | 0.04 | 7.62 | <2e-16 |  |
| PD | 1.60 | 0.03 | 50.04 | <2e-16 |  |
| PD | -1.39 | 0.07 | -20.46 | <2e-16 |  |
| **Smooth terms** | **edf** | **Ref.df** | **Chi.sq** | **P** |  |
| s(AGE) | 6.40 | 7.42 | 186.41 | <2e-16 |  |
| s(AGE): MD | 1.01 | 1.03 | 34.58 | 0.00 |  |
| s(AGE): DYS | 1.36 | 1.64 | 11.29 | 0.01 |  |
| s(AGE): GAD | 2.78 | 3.50 | 15.82 | 0.00 |  |
| s(AGE): PD | 4.62 | 5.64 | 13.76 | 0.03 |  |

*Chi.sq, Chi-squared test value; DYS, dysthymia; edf, effective degrees of freedom; GAD, generalized anxiety disorder; MD, major depression; P, p-value; PD, panic disorder; Ref.df, reference number of effective degrees of freedom used for hypothesis testing; SPH, social phobia; SE, standard error; z, z-test value.*

*Parametric and smooth terms of GAMs describing the prevalence of all internalizing disorders by age, and allowing a separate smooth for each internalizing disorder. The first rows represent the parametric and smooth terms of all internalizing disorders compared with MD (reference class). The following rows represent the smooth terms for a different reference class (in bold). The smooth terms indicate whether the development over age is significantly different for each disorder. The ‘edf’ reflects the number of effective degrees of freedom, which is an estimate of how many parameters are needed to represent the smooth. An edf>1 indicates a nonlinear smooth. The P-value associated with the smooth term indicates if the smooth is significantly different from 0. The results indicate that in 8 out of 10 pairwise comparisons, the smooth terms for each disorder are consistently significantly different. Only the smooth terms for PD-SPH and GAD-DYS are not consistently significantly different from each other, as indicated by when changing the reference class (NS). Note that the parametric terms are consistently significantly different, regardless of which disorder is chosen as the reference class.*

**SUPPLEMENTAL TABLE 3. Generalized additive models testing differences between men and women**

| **MD** | **Estimate** | **SE** | **z** | **P** |
| --- | --- | --- | --- | --- |
| (Intercept) | -4.15 | 0.03 | -125.18 | <2e-16 |
| Sex(Female) | 0.56 | 0.04 | 14.15 | <2e-16 |
| **Smooth function** | **edf** | **Ref.df** | **Chi.sq** | **P** |
| s(Age) | 4.62 | 5.64 | 42.59 | <0.01 |
| s(Age):Sex | 2.58 | 3.23 | 6.54 | 0.10 |
| **DYS** | **Estimate** | **SE** | **z** | **P** |
| (Intercept) | -4.80 | 0.05 | -105.24 | <2e-16 |
| Sex(Female) | 0.52 | 0.05 | 9.53 | <2e-16 |
| **Smooth function** | **edf** | **Ref.df** | **Chi.sq** | **P** |
| s(Age) | 4.49 | 5.52 | 44.30 | <0.01 |
| s(Age):Sex | 1.00 | 1.00 | 0.89 | 0.35 |
| **GAD** | **Estimate** | **SE** | **z** | **P** |
| (Intercept) | -3.48 | 0.02 | -142.50 | <2e-16 |
| Sex(Female) | 0.53 | 0.03 | 18.19 | <2e-16 |
| **Smooth function** | **edf** | **Ref.df** | **Chi.sq** | **P** |
| s(Age) | 5.75 | 6.80 | 148.85 | <2e-16 |
| s(Age):Sex | 2.53 | 3.17 | 4.32 | 0.23 |
| **PD** | **Estimate** | **SE** | **z** | **P** |
| (Intercept) | -6.54 | 0.11 | -57.80 | <2e-16 |
| Sex(Female) | 0.62 | 0.13 | 4.82 | <0.01 |
| **Smooth function** | **edf** | **Ref.df** | **Chi.sq** | **P** |
| s(Age) | 5.11 | 6.16 | 34.92 | <0.01 |
| s(Age):Sex | 1.20 | 1.36 | 1.55 | 0.24 |
| **SPH** | **Estimate** | **SE** | **t** | **P** |
| (Intercept) | -4.86 | 0.05 | -102.83 | <2e-16 |
| Sex(Female) | 0.21 | 0.06 | 3.59 | <0.01 |
| **Smooth function** | **edf** | **Ref.df** | **Chi.sq** | **P** |
| s(Age) | 4.69 | 5.72 | 39.06 | <0.01 |
| s(Age):Sex | 2.28 | 2.86 | 1.66 | 0.67 |
| **MD symptoms** | **Estimate** | **SE** | **t** | **P** |
| (Intercept) | 0.41 | 0.01 | 59.63 | <2e-16 |
| Sex(Female) | 0.24 | 0.01 | 27.16 | <2e-16 |
| **Smooth function** | **edf** | **Ref.df** | **F** | **P** |
| s(Age) | 6.10 | 7.24 | 6.92 | 2.12e-08 |
| s(Age):Sex | 1.00 | 1.00 | 0.84 | 0.36* |
| **GAD symptoms** | **Estimate** | **SE** | **t** | **P** |
| (Intercept) | 0.84 | 0.01 | 80.87 | <2e-16 |
| Sex(Female) | 0.48 | 0.01 | 35.02 | <2e-16 |
| **Smooth function** | **edf** | **Ref.df** | **F** | **P** |
| s(Age) | 6.98 | 8.01 | 34.66 | <2e-16 |
| s(Age):Sex | 1.00 | 1.01 | 10.20 | <0.01* |
| **Negative Affect** | **Estimate** | **SE** | **t** | **P** |
| (Intercept) | 19.64 | 0.02 | 900.64 | <2e-16 |
| Sex(Female) | 1.83 | 0.03 | 64.28 | <2e-16 |
| **Smooth function** | **edf** | **Ref.df** | **F** | **P** |
| s(Age) | 6.14 | 7.20 | 25.04 | <2e-16 |
| s(Age):Sex | 4.12 | 5.06 | 4.24 | <0.01 |
| **Neuroticism** | **Estimate** | **SE** | **t** | **P** |
| (Intercept) | 115.04 | 0.16 | 738.41 | <2e-16 |
| Sex(Female) | 8.55 | 0.20 | 41.91 | <2e-16 |
| **Smooth function** | **edf** | **Ref.df** | **F** | **P** |
| s(Age) | 2.93 | 3.64 | 30.56 | <2e-16 |
| s(Age):Sex | 2.78 | 3.44 | 7.22 | <0.01 |

*Chi.sq, Chi-squared test value; DYS, dysthymia; edf, effective degrees of freedom; F, F-test value; GAD, generalized anxiety disorder; MD, major depression; P, p-value; PD, panic disorder; Ref.df, reference number of effective degrees of freedom used for hypothesis testing; SE, standard error; SPH, social phobia, SE, standard deviation; t, t-test value; z, z-test value.*

*Parametric and smooth terms of generalized additive models describing each internalizing disorder or trait using age and sex as independent variables, and allowing a separate smooth for men and women. The reference class for sex are men. The ‘edf’ reflects the number of effective degrees of freedom, which is an estimate of how many parameters are needed to represent the smooth. An edf>1 indicates a nonlinear smooth. The P-value associated with the smooth term indicates if the smooth is significantly different from 0.(1) Although the intercepts of the GAMs were clearly significantly different across the internalizing disorders, the curves over age were similar for men and women. In other words, the difference in logits (or log-odds=log(p/(1-p)) in prevalence remained constant over the lifespan. This means that internalizing disorders occur more often in men than in women, but the relative difference in prevalence between men and women remained stable across different ages.*

**The smooth curve of the difference between men and women is significant for GAD symptoms, but not MD symptoms, even though the edf values are the same due to rounding. The edf and Ref.df values are 1.004 and 1.008 for GAD and 1.002 and 1.003 for MD, respectively.*

**SUPPLEMENTAL TABLE 4. Internalizing disorders and traits over lifetime for men and women estimated by generalized additive models**

| **Age (years)** | **18-24** | **25-34** | **35-44** | **45-54** | **55-64** | **65-74** | **75-80** |
| --- | --- | --- | --- | --- | --- | --- | --- |
| **MD, N (N complete)** | **10858 (10858)** | **25987 (25987)** | **42527 (42526)** | **39219 (39219)** | **19205 (19205)** | **7630 (7630)** | **889 (889)** |
| Total, % (SE) | 2.22 (0.14) | 2.26 (0.09) | 2.39 (0.07) | 2.39 (0.08) | 1.84 (0.10) | 1.36 (0.13) | 1.02 (0.34) |
| Men, % (SE) | 1.52 (0.20) | 1.60 (0.12) | 1.68 (0.10) | 1.69 (0.10) | 1.31 (0.13) | 0.99 (0.16) | 0.74 (0.42) |
| Women, % (SE) | 2.59 (0.19) | 2.74 (0.13) | 2.87 (0.11) | 2.89 (0.11) | 2.25 (0.14) | 1.70 (0.20) | 1.27 (0.52) |
| Risk ratio (SE) | 1.71 (1.16) | 1.71 (1.09) | 1.70 (1.07) | 1.70 (1.07) | 1.72 (1.12) | 1.72 (1.23) | 1.72 (2.01) |
| **DYS, N (N complete)** | **10858 (10598)** | **25987 (25302)** | **42527 (41365)** | **39219 (38109)** | **19205 (18794)** | **7630 (7504)** | **889 (877)** |
| Total, % (SE) | 4.63 (0.20) | 5.07 (0.14) | 4.82 (0.10) | 4.46 (0.10) | 2.75 (0.12) | 1.56 (0.14) | 0.98 (0.33) |
| Men, % (SE) | 3.26 (0.29) | 3.70 (0.18) | 3.49 (0.14) | 3.24 (0.14) | 1.98 (0.15) | 1.16 (0.18) | 0.72 (0.42) |
| Women, % (SE) | 5.34 (0.27) | 6.07 (0.19) | 5.74 (0.15) | 5.33 (0.15) | 3.33 (0.17) | 1.94 (0.22) | 1.21 (0.50) |
| Risk ratio (SE) | 1.64 (1.11) | 1.64 (1.06) | 1.64 (1.05) | 1.64 (1.05) | 1.68 (1.10) | 1.68 (1.21) | 1.68 (2.03) |
| **GAD, N (N complete)** | **10858 (10858)** | **25987 (25987)** | **42527 (42527)** | **39219 (39219)** | **19205 (19205)** | **7630 (7630)** | **889 (889)** |
| Total, % (SE) | 1.30 (0.11) | 1.31 (0.07) | 1.27 (0.05) | 1.27 (0.06) | 0.88 (0.07) | 0.49 (0.08) | 0.30 (0.18) |
| Men, % (SE) | 0.90 (0.16) | 0.94 (0.09) | 0.91 (0.07) | 0.91 (0.07) | 0.63 (0.09) | 0.36 (0.10) | 0.22 (0.23) |
| Women, % (SE) | 1.51 (0.14) | 1.57 (0.10) | 1.52 (0.08) | 1.53 (0.08) | 1.07 0.10) | 0.61 (0.12) | 0.37 (0.28) |
| Risk ratio (SE) | 1.68 (1.22) | 1.68 (1.12) | 1.68 (1.10) | 1.68 (1.10) | 1.70 (1.18) | 1.70 (1.41) | 1.70 (3.65) |
| **PD, N (N complete)** | **10858 (10858)** | **25987 (25987)** | **42527 (42527)** | **39219 (39219)** | **19205 (19205)** | **7630 (7630)** | **889 (889)** |
| Total, % (SE) | 0.28 (0.05) | 0.39 (0.04) | 0.26 (0.02) | 0.21 (0.02) | 0.14 (0.03) | 0.05 (0.02) | 0.02 (0.05) |
| Men, % (SE) | 0.19 (0.07) | 0.27 (0.05) | 0.18 (0.03) | 0.14 (0.03) | 0.09 (0.03) | 0.03 (0.03) | 0.01 (0.06) |
| Women, % (SE) | 0.33 (0.07) | 0.47 (0.06) | 0.32 (0.04) | 0.25 (0.03) | 0.17 (0.04) | 0.06 (0.04) | 0.02 (0.07) |
| Risk ratio (SE) | 1.72 (1.53) | 1.74 (1.24) | 1.74 (1.23) | 1.74 (1.28) | 1.78 (1.53) | 1.76 (3.04) | N.A. |
| **SPH, N (N complete)** | **10858 (10858)** | **25987 (25987)** | **42527 (42526)** | **39219 (39219)** | **19205 (19205)** | **7630 (7629)** | **889 (889)** |
| Total, % (SE) | 1.18 0.10) | 1.22 (0.07) | 0.95 (0.05) | 0.86 (0.05) | 0.58 (0.05) | 0.37 (0.07) | 0.30 (0.18) |
| Men, % (SE) | 1.02 (0.17) | 1.08 (0.10) | 0.83 (0.07) | 0.75 (0.07) | 0.51 (0.08) | 0.33 (0.09) | 0.26 (0.25) |
| Women, % (SE) | 1.26 (0.13) | 1.33 (0.09) | 1.03 (0.06) | 0.93 (0.06) | 0.64 (0.08) | 0.41 (0.10) | 0.33 (0.26) |
| Risk ratio (SE) | 1.24 (1.21) | 1.24 (1.12) | 1.24 (1.11) | 1.24 (1.12) | 1.26 (1.22) | 1.24 (1.46) | 1.24 (3.47) |
| **MD symptoms,**  **N (N complete)** | **10858 (6722)** | **25987 (13694)** | **42527 (20982)** | **39219 (20238)** | **19205 (10993)** | **7630 (4504)** | **889 (533)** |
| Total, m (sd) | 0.58 (0.12) | 0.55 (0.12) | 0.55 (0.12) | 0.58 (0.12) | 0.52 (0.12) | 0.47 (0.12) | 0.52 (0.12) |
| Men, m (sd) | 0.42 (<0.01) | 0.41 (0.01) | 0.41 (0.01) | 0.44 (0.01) | 0.38 (0.03) | 0.34 (0.01) | 0.39 (0.01) |
| Women, m (sd) | 0.66 (<0.01) | 0.65 (0.01) | 0.65 (0.01) | 0.68 (0.01) | 0.62 (0.03) | 0.58 (0.01) | 0.63 (0.01) |
| Mean ratio (SE) | 1.58 (1.00) | 1.60 (1.00) | 1.60 (1.00) | 1.55 (1.00) | 1.65 (1.00) | 1.72 (1.00) | 1.62 (1.00) |
| **GAD symptoms,**  **N (N complete)** | **10858 (6698)** | **25987 (13767)** | **42527 (2960)** | **39219 (20125)** | **19205 (10827)** | **7630 (4423)** | **889 (514)** |
| Total, m (sd) | 1.19 (0.26) | 1.24 (0.26) | 1.19 (0.24) | 1.16 (0.23) | 0.93 (0.24) | 0.71 (0.20) | 0.71 (0.18) |
| Men, m (sd) | 0.83 (0.05) | 0.94 (0.01) | 0.90 (0.01) | 0.89 (0.02) | 0.68 (0.09) | 0.50 (0.01) | 0.52 (0.00) |
| Women, m (sd) | 1.38 (0.04) | 1.46 (0.01) | 1.39 (0.02) | 1.36 (0.02) | 1.11 (0.10) | 0.90 (0.01) | 0.89 (0.01) |
| Mean ratio (SE) | 1.66 (1.00) | 1.56 (1.00) | 1.54 (1.00) | 1.52 (1.00) | 1.64 (1.00) | 1.79 (1.00) | 1.71 (1.00) |
| **Neuroticism,**  **N (N complete)** | **10858 (4050)** | **25987 (7958)** | **42527 (12281)** | **39219 (12032)** | **19205 (6297)** | **7630 (37)** | **889 (<10)** |
| Total, m (sd) | 125.61 (5.09) | 122.41 (4.85) | 119.79 (4.15) | 118.39 (3.82) | 117.72 (3.88) | 117.04 (3.97) | 116.84 (4.03) |
| Men, m (sd) | 118.56 (0.46) | 116.80 (0.59) | 114.86 (0.45) | 113.86 (0.19) | 113.26 (0.12) | 112.89 (0.12) | 112.55 (0.05) |
| Women, m (sd) | 129.24 (0.66) | 126.48 (0.98) | 123.20 (0.77) | 121.61 (0.23) | 121.09 (0.08) | 120.84 (0.07) | 120.62 (0.03) |
| Mean ratio (SE) | 1.09 (1.00) | 1.08 (1.00) | 1.07 (1.00) | 1.07 (1.00) | 1.07 (1.00) | 1.07 (1.00) | 1.07 (1.00) |
| **Negative affect,**  **N (N complete)** | **10858 (10125)** | **25987 (24178)** | **42527 (40414)** | **39219 (37649)** | **19205 (18537)** | **7630 (7163)** | **889 (793)** |
| Total, m (sd) | 21.41 (0.91) | 21.01 (0.92) | 20.67 (0.86) | 20.61 (0.87) | 20.51 (1.01) | 20.08 (1.05) | 19.91 (1.06) |
| Men, m (sd) | 20.15 (0.07) | 19.94 (0.07) | 19.64 (0.07) | 19.58 (0.02) | 19.35 (0.15) | 18.98 (0.07) | 18.79 (0.04) |
| Women, m (sd) | 22.06 (0.09) | 21.79 (0.09) | 21.37 (0.10) | 21.35 (0.07) | 21.38 (0.10) | 21.08 (0.06) | 20.90 (0.04) |
| Mean ratio (SE) | 1.09 (1.00) | 1.09 (1.00) | 1.09 (1.00) | 1.09 (1.00) | 1.10 (1.00) | 1.11 (1.00) | 1.11 (1.00) |

*DYS, dysthymia; GAD, generalized anxiety disorder; MD, major depression; N, number of subjects in the GAM model; N, number of subjects in this age group; N complete, number of subjects with complete phenotype data in this age group; PD, panic disorder; sd, standard deviation; SE, standard error; SPH, social phobia.*

*Point prevalence of internalizing disorders and means of internalizing traits in each 10-year age bin calculated from the predictions based on GAM models, in all subjects in this age group (N). Note that in Supplementary Table 3, the models for GAD sumscore, neuroticism and negative affect had different intercepts and slopes for both genders. The models for all internalizing disorders (MD, DYS, GAD, PD, SPH) and the MD symptoms had significantly different intercepts but not slopes for men and women. For these outcomes, this table is based on models without an interaction term. The risk and mean ratios are mostly stable over age. Note that because of the low prevalence rates the odds ratio and the relative risk are nearly identical. N. B. The standard erros of the mean ratios of the internalizing symptoms are not exactly the same for all age bins, but the differences only occur on the fifth decimal.* **SUPPLEMENTAL TABLE 5. Generalized additive models testing differences between internalizing disorders with random intercepts in 10% subsamples (n=14,624)**

|  | **Without random intercept** | | | | **With random intercept** | | | |  |
| --- | --- | --- | --- | --- | --- | --- | --- | --- | --- |
| **Sample 1** | **Estimate** | **SE** | **z** | **P** | **Estimate** | **SE** | **z** | **P** |  |
| (Intercept: MD) | -3.96 | 0.08 | -50.06 | <2e-16 | -4.17 | 0.08 | -51.61 | <2e-16 |  |
| DYS | -0.61 | 0.13 | -4.51 | <0.01 | -0.47 | 0.13 | -3.61 | <0.01 |  |
| GAD | 0.53 | 0.08 | 6.51 | <0.01 | 0.55 | 0.09 | 5.90 | <0.01 |  |
| PD | -1.28 | 0.14 | -9.32 | <2e-16 | -1.32 | 0.14 | -9.55 | <2e-16 |  |
| SPH | -1.01 | 0.12 | -8.26 | <2e-16 | -1.05 | 0.12 | -8.47 | <2e-16 |  |
| **Smooth terms** | **edf** | **Ref.df** | **Chi.sq** | **P** | **edf** | **Ref.df** | **Chi.sq** | **P** |  |
| s(AGE) | 4.67 | 5.71 | 25.24 | <0.01 | 3.35 | 4.05 | 8.76 | 0.07 |  |
| s(AGE): DYS | 2.09 | 2.64 | 1.73 | 0.41 | 1.79 | 2.24 | 0.91 | 0.57 |  |
| s(AGE): GAD | 1.07 | 1.14 | 4.91 | 0.03 | 1.46 | 1.78 | 6.02 | 0.04 |  |
| s(AGE): PD | 1.00 | 1.00 | 4.91 | 0.03 | 1.00 | 1.00 | 4.76 | 0.03 |  |
| s(AGE): SPH | 1.00 | 1.00 | 1.23 | 0.27 | 1.00 | 1.00 | 1.15 | 0.28 |  |
| s(ID) |  |  |  |  | 1994.03 | 14622.00 | 3575.33 | <2e-16 |  |
| **Sample 2** | **Estimate** | **SE** | **z** | **P** | **Estimate** | **SE** | **z** | **P** |  |
| (Intercept: MD) | -3.95 | 0.08 | -49.31 | <2e-16 | -4.15 | 0.08 | -52.26 | <2e-16 |  |
| DYS | -0.88 | 0.15 | -5.89 | <0.01 | -0.73 | 0.15 | -4.82 | <0.01 |  |
| GAD | 0.52 | 0.10 | 5.26 | <0.01 | 0.57 | 0.10 | 5.79 | <0.01 |  |
| PD | -1.48 | 0.15 | -9.92 | <2e-16 | -1.53 | 0.15 | -10.11 | <2e-16 |  |
| SPH | -0.95 | 0.19 | -5.04 | <0.01 | -0.94 | 0.12 | -7.88 | <0.01 |  |
| **Smooth terms** | **edf** | **Ref.df** | **Chi.sq** | **P** | **edf** | **Ref.df** | **Chi.sq** | **P** |  |
| s(AGE) | 3.62 | 4.51 | 11.48 | 0.03 | 2.99 | 3.64 | 8.17 | 0.07 |  |
| s(AGE): DYS | 1.92 | 2.42 | 2.88 | 0.29 | 2.00 | 2.52 | 3.44 | 0.25 |  |
| s(AGE): GAD | 2.03 | 2.56 | 7.52 | 0.05 | 1.91 | 2.39 | 7.78 | 0.03 |  |
| s(AGE): PD | 1.00 | 1.00 | 13.90 | <0.01 | 1.00 | 1.00 | 14.22 | <0.01 |  |
| s(AGE): SPH | 3.38 | 4.23 | 11.67 | 0.02 | 1.00 | 1.00 | 6.81 | 0.01 |  |
| s(ID) |  |  |  |  | 1968.96 | 14622.00 | 433.52 | <2e-16 |  |
| **Sample 3** | **Estimate** | **SE** | **z** | **P** | **Estimate** | **SE** | **z** | **P** |  |
| (Intercept: MD) | -3.97 | 0.08 | -50.45 | <2e-16 | -4.17 | 0.08 | -51.63 | <2e-16 |  |
| DYS | -0.55 | 0.10 | -5.28 | <0.01 | -0.40 | 0.11 | -3.78 | <0.01 |  |
| GAD | 0.55 | 0.09 | 5.96 | <0.01 | 0.58 | 0.10 | 6.09 | <0.01 |  |
| PD | -1.52 | 0.15 | -9.99 | <2e-16 | -1.57 | 0.15 | -10.17 | <2e-16 |  |
| SPH | -1.06 | 0.13 | -8.43 | <2e-16 | -1.10 | 0.13 | -8.60 | <2e-16 |  |
| **Smooth terms** | **edf** | **Ref.df** | **Chi.sq** | **P** | **edf** | **Ref.df** | **Chi.sq** | **P** |  |
| s(AGE) | 4.26 | 5.26 | 23.40 | <0.01 | 3.60 | 4.35 | 14.56 | 0.01 |  |
| s(AGE): DYS | 1.00 | 1.01 | 1.07 | 0.30 | 1.00 | 1.00 | 1.37 | 0.24 |  |
| s(AGE): GAD | 1.59 | 1.98 | 6.94 | 0.03 | 1.65 | 2.06 | 7.90 | 0.02 |  |
| s(AGE): PD | 1.00 | 1.00 | 9.36 | <0.01 | 1.00 | 1.00 | 9.20 | <0.01 |  |
| s(AGE): SPH | 1.00 | 1.00 | 5.52 | 0.02 | 1.00 | 1.00 | 5.42 | 0.02 |  |
| s(ID) |  |  |  |  | 1950.21 | 14622.00 | 3350.11 | <2e-16 |  |
| **Sample4** | **Estimate** | **SE** | **z** | **P** | **Estimate** | **SE** | **z** | **P** |  |
| (Intercept: MD) | -4.09 | 0.08 | -5<0.01 | <2e-16 | -4.30 | 0.08 | -51.23 | <2e-16 |  |
| DYS | -0.72 | 0.11 | -6.44 | <0.01 | -0.53 | 0.11 | -4.68 | <0.01 |  |
| GAD | 0.61 | 0.08 | 7.73 | <0.01 | 0.65 | 0.08 | 8.03 | <0.01 |  |
| PD | -1.57 | 0.15 | -10.36 | <2e-16 | -1.63 | 0.15 | -10.59 | <2e-16 |  |
| SPH | -1.19 | 0.13 | -9.18 | <2e-16 | -1.24 | 0.13 | -9.40 | <2e-16 |  |
| **Smooth terms** | **edf** | **Ref.df** | **Chi.sq** | **P** | **edf** | **Ref.df** | **Chi.sq** | **P** |  |
| s(AGE) | 4.49 | 5.52 | 50.25 | <0.01 | 3.90 | 4.71 | 33.67 | <0.01 |  |
| s(AGE): DYS | 1.00 | 1.00 | 5.16 | 0.02 | 1.00 | 1.00 | 4.74 | 0.03 |  |
| s(AGE): GAD | 1.00 | 1.00 | 3.00 | 0.08 | 1.00 | 1.00 | 3.21 | 0.07 |  |
| s(AGE): PD | 1.00 | 1.00 | 2.94 | 0.09 | 1.00 | 1.00 | 3.07 | 0.08 |  |
| s(AGE): SPH | 1.00 | 1.00 | 2.35 | 0.13 | 1.00 | 1.00 | 2.45 | 0.12 |  |
| s(ID) |  |  |  |  | 1941.77 | 14622.00 | 3383.93 | <2e-16 |  |
| **Sample 5** | **Estimate** | **SE** | **z** | **P** | **Estimate** | **SE** | **z** | **P** |  |
| (Intercept: MD) | -3.77 | 0.07 | -54.59 | <2e-16 | -4.00 | 0.07 | -55.12 | <2e-16 |  |
| DYS | -0.82 | 0.11 | -7.77 | <0.01 | -0.66 | 0.11 | -6.12 | <0.01 |  |
| GAD | 0.54 | 0.07 | 7.23 | <0.01 | 0.57 | 0.08 | 7.47 | <0.01 |  |
| PD | -1.55 | 0.21 | -7.50 | <0.01 | -1.32 | 0.13 | -10.30 | <2e-16 |  |
| SPH | -1.02 | 0.11 | -9.04 | <2e-16 | -1.06 | 0.12 | -9.23 | <2e-16 |  |
| **Smooth terms** | **edf** | **Ref.df** | **Chi.sq** | **P** | **edf** | **Ref.df** | **Chi.sq** | **P** |  |
| s(AGE) | 5.16 | 6.25 | 28.75 | <0.01 | 4.14 | 4.96 | 20.19 | <0.01 |  |
| s(AGE): DYS | 1.00 | 1.00 | 0.17 | 0.68 | 1.00 | 1.00 | 0.28 | 0.60 |  |
| s(AGE): GAD | 1.00 | 1.00 | 3.73 | 0.05 | 1.00 | 1.00 | 4.27 | 0.04 |  |
| s(AGE): PD | 2.26 | 2.87 | 7.37 | 0.06 | 1.00 | 1.00 | 4.10 | 0.04 |  |
| s(AGE): SPH | 1.00 | 1.00 | 1.04 | 0.31 | 1.00 | 1.00 | 1.00 | 0.32 |  |
| s(ID) |  |  |  |  | 2043.33 | 14622.00 | 3525.23 | <2e-16 |  |
| **Sample 6** | **Estimate** | **SE** | **z** | **P** | **Estimate** | **SE** | **z** | **P** |  |
| (Intercept: MD) | -4.04 | 0.08 | -52.01 | <2e-16 | -4.28 | 0.08 | -52.37 | <2e-16 |  |
| DYS | -0.51 | 0.11 | -4.65 | <0.01 | -0.33 | 0.11 | -2.99 | <0.01 |  |
| GAD | 0.64 | 0.08 | 7.74 | <0.01 | 0.68 | 0.09 | 8.02 | <0.01 |  |
| PD | -1.43 | 0.15 | -9.38 | <2e-16 | -1.48 | 0.15 | -9.60 | <2e-16 |  |
| SPH | -0.96 | 0.13 | -7.55 | <0.01 | -1.00 | 0.13 | -7.73 | <0.01 |  |
| **Smooth terms** | **edf** | **Ref.df** | **Chi.sq** | **P** | **edf** | **Ref.df** | **Chi.sq** | **P** |  |
| s(AGE) | 3.80 | 4.73 | 29.38 | <0.01 | 3.28 | 3.98 | 19.66 | <0.01 |  |
| s(AGE): DYS | 1.00 | 1.00 | 0.18 | 0.67 | 1.00 | 1.00 | 0.16 | 0.69 |  |
| s(AGE): GAD | 1.00 | 1.00 | 1.44 | 0.23 | 1.00 | 1.00 | 1.63 | 0.20 |  |
| s(AGE): PD | 1.00 | 1.00 | 1.57 | 0.21 | 1.00 | 1.00 | 1.56 | 0.21 |  |
| s(AGE): SPH | 1.00 | 1.00 | 2.43 | 0.12 | 1.00 | 1.00 | 2.45 | 0.12 |  |
| s(ID) |  |  |  |  | 1933.10 | 14622.00 | 3391.28 | <2e-16 |  |
| **Sample 7** | **Estimate** | **SE** | **z** | **P** | **Estimate** | **SE** | **z** | **P** |  |
| (Intercept: MD) | -3.95 | 0.08 | -50.59 | <2e-16 | -4.17 | 0.08 | -51.88 | <2e-16 |  |
| DYS | -0.62 | 0.11 | -5.89 | <0.01 | -0.44 | 0.11 | -4.12 | <0.01 |  |
| GAD | 0.47 | 0.10 | 4.53 | <0.01 | 0.50 | 0.11 | 4.66 | <0.01 |  |
| PD | -1.36 | 0.14 | -9.71 | <2e-16 | -1.42 | 0.14 | -9.98 | <2e-16 |  |
| SPH | -1.00 | 0.12 | -8.24 | <2e-16 | -1.04 | 0.13 | -8.14 | <0.01 |  |
| **Smooth terms** | **edf** | **Ref.df** | **Chi.sq** | **P** | **edf** | **Ref.df** | **Chi.sq** | **P** |  |
| s(AGE) | 3.65 | 4.56 | 20.44 | <0.01 | 2.98 | 3.63 | 10.73 | 0.02 |  |
| s(AGE): DYS | 1.00 | 1.00 | 0.01 | 0.92 | 1.00 | 1.00 | 0.06 | 0.80 |  |
| s(AGE): GAD | 2.22 | 2.80 | 4.79 | 0.16 | 2.31 | 2.91 | 5.69 | 0.12 |  |
| s(AGE): PD | 1.01 | 1.01 | 3.32 | 0.07 | 1.00 | 1.00 | 3.22 | 0.07 |  |
| s(AGE): SPH | 1.00 | 1.00 | 2.35 | 0.12 | 1.08 | 1.15 | 1.79 | 0.17 |  |
| s(ID) |  |  |  |  | 2031.73 | 14622.00 | 3647.21 | <2e-16 |  |
| **Sample 8** | **Estimate** | **SE** | **z** | **P** | **Estimate** | **SE** | **z** | **P** |  |
| (Intercept: MD) | -3.93 | 0.07 | -53.29 | <2e-16 | -4.14 | 0.08 | -53.58 | <2e-16 |  |
| DYS | -0.96 | 0.12 | -8.15 | <0.01 | -0.80 | 0.12 | -6.68 | <0.01 |  |
| GAD | 0.59 | 0.08 | 7.58 | <0.01 | 0.62 | 0.08 | 7.84 | <0.01 |  |
| PD | -1.28 | 0.13 | -9.54 | <2e-16 | -1.32 | 0.14 | -9.73 | <2e-16 |  |
| SPH | -1.06 | 0.13 | -7.90 | <0.01 | -1.08 | 0.14 | -7.64 | <0.01 |  |
| **Smooth terms** | **edf** | **Ref.df** | **Chi.sq** | **P** | **edf** | **Ref.df** | **Chi.sq** | **P** |  |
| s(AGE) | 3.89 | 4.84 | 31.33 | <0.01 | 3.53 | 4.27 | 22.64 | <0.01 |  |
| s(AGE): DYS | 1.00 | 1.00 | 1.70 | 0.19 | 1.00 | 1.00 | 1.64 | 0.20 |  |
| s(AGE): GAD | 1.00 | 1.00 | 3.13 | 0.08 | 1.00 | 1.00 | 3.50 | 0.06 |  |
| s(AGE): PD | 1.00 | 1.00 | 6.96 | 0.01 | 1.00 | 1.00 | 7.03 | 0.01 |  |
| s(AGE): SPH | 1.24 | 1.45 | 5.35 | 0.11 | 1.38 | 1.67 | 5.82 | 0.12 |  |
| s(ID) |  |  |  |  | 1943.24 | 14622.00 | 3340.06 | <2e-16 |  |
|  |  |  |  |  |  |  |  |  |  |
| **Sample 9** | **Estimate** | **SE** | **z** | **P** | **Estimate** | **SE** | **z** | **P** |  |
| (Intercept: MD) | -3.88 | 0.06 | -61.88 | <2e-16 | -4.11 | 0.07 | -62.00 | <2e-16 |  |
| DYS | -0.79 | 0.13 | -6.00 | <0.01 | -0.61 | 0.13 | -4.80 | <0.01 |  |
| GAD | 0.44 | 0.11 | 4.15 | <0.01 | 0.51 | 0.11 | 4.84 | <0.01 |  |
| PD | -1.50 | 0.21 | -7.30 | <0.01 | -1.53 | 0.21 | -7.43 | <0.01 |  |
| SPH | -0.93 | 0.14 | -6.73 | <0.01 | -0.94 | 0.14 | -6.59 | <0.01 |  |
| **Smooth terms** | **edf** | **Ref.df** | **Chi.sq** | **P** | **edf** | **Ref.df** | **Chi.sq** | **P** |  |
| s(AGE) | 1.00 | 1.00 | 0.09 | 0.76 | 1.00 | 1.00 | 0.47 | 0.49 |  |
| s(AGE): DYS | 1.75 | 2.20 | 4.73 | 0.11 | 1.51 | 1.86 | 4.21 | 0.10 |  |
| s(AGE): GAD | 3.69 | 4.60 | 22.48 | <0.01 | 3.17 | 3.93 | 21.80 | <0.01 |  |
| s(AGE): PD | 2.23 | 2.82 | 11.52 | 0.01 | 2.13 | 2.69 | 11.49 | 0.01 |  |
| s(AGE): SPH | 1.80 | 2.26 | 7.80 | 0.02 | 1.95 | 2.44 | 8.41 | 0.02 |  |
| s(ID) |  |  |  |  | 1913.74 | 14622.00 | 3300.91 | <2e-16 |  |
| **Sample 10** | **Estimate** | **SE** | **z** | **P** | **Estimate** | **SE** | **z** | **P** |  |
| (Intercept: MD) | -4.19 | 0.09 | -44.88 | <2e-16 | -4.40 | 0.09 | -46.39 | <2e-16 |  |
| DYS | -0.51 | 0.11 | -4.52 | <0.01 | -0.34 | 0.12 | -2.98 | <0.01 |  |
| GAD | 0.67 | 0.11 | 5.95 | <0.01 | 0.70 | 0.12 | 6.00 | <0.01 |  |
| PD | -1.59 | 0.36 | -4.39 | <0.01 | -1.67 | 0.38 | -4.39 | <0.01 |  |
| SPH | -1.02 | 0.14 | -7.42 | <0.01 | -1.07 | 0.14 | -7.59 | <0.01 |  |
| **Smooth terms** | **edf** | **Ref.df** | **Chi.sq** | **P** | **edf** | **Ref.df** | **Chi.sq** | **P** |  |
| s(AGE) | 3.73 | 4.64 | 26.02 | <0.01 | 3.11 | 3.79 | 17.13 | <0.01 |  |
| s(AGE): DYS | 1.00 | 1.00 | 1.19 | 0.28 | 1.00 | 1.00 | 0.71 | 0.40 |  |
| s(AGE): GAD | 2.07 | 2.62 | 2.44 | 0.36 | 2.21 | 2.78 | 3.40 | 0.26 |  |
| s(AGE): PD | 4.09 | 5.04 | 13.02 | 0.02 | 4.18 | 5.13 | 13.28 | 0.02 |  |
| s(AGE): SPH | 1.00 | 1.00 | 4.25 | 0.04 | 1.00 | 1.00 | 4.13 | 0.04 |  |
| s(ID) |  |  |  |  | 1987.96 | 14622.00 | 3555.73 | <2e-16 |  |
|  |  |  |  |  |  |  |  |  |  |

*Chi.sq, Chi-squared test value; DYS, dysthymia; edf, effective degrees of freedom; GAD, generalized anxiety disorder; ID, identifier for each subject; MD, major depression; P, p-value; PD, panic disorder; edf, effective degrees of freedom; Ref.df, reference number of effective degrees of freedom used for hypothesis testing; SE, standard error; SPH, social phobia, z, z-test value.*

*Parametric and smooth terms of GAMs in 10% random subsamples excluding (left set of columns) and including (right set of columns) random intercepts for each subject. The first rows represent the parametric terms of all internalizing disorders compared with MD (reference class). The following rows represent the smooth terms that indicate whether the development over age is significantly different for each disorder. The ‘edf’ reflects the number of effective degrees of freedom, which is an estimate of how many parameters are needed to represent the smooth. An edf>1 indicates a nonlinear smooth. The P-value associated with the smooth term indicates if the smooth is significantly different from 0.*

*The results show that in all 10 random effect models the random intercepts were highly significant. The models including a randam intercept explained more variance than the models excluding random intercepts (adj. R2 ~25% vs ~1%), and improved model fit. This indicated that there was individual variation in susceptibility for internalizing disorders. However, the estimated trajectories of prevalence of internalizing disorders over lifetime were similar in all models including and excluding random intercepts. This indicated that the effect of age on the point prevalence could not be explained by individuals with a high susceptibility for internalizing disorders being overrepresented in certain age groups.*

**SUPPLEMENTAL TABLE 6. Generalized additive models testing differences testing differences between men and women, without and with random intercepts for family membership.**

|  | **Without random intercept** | | | | **With random intercept** | | | |  |
| --- | --- | --- | --- | --- | --- | --- | --- | --- | --- |
| **MD** | **Estimate** | **SE** | **z** | **P** | **Estimate** | **SE** | **z** | **P** |  |
| (Intercept) | -4.15 | 0.03 | -125.18 | <2e-16 | -3.76 | 0.05 | -76.17 | <2e-16 |  |
| Sex(Female) | 0.56 | 0.04 | 14.15 | <2e-16 | 0.56 | 0.04 | 14.21 | <2e-16 |  |
| **Smooth function** | **edf** | **Ref.df** | **Chi.sq** | **P** | **edf** | **Ref.df** | **Chi.sq** | **P** |  |
| s(Age) | 4.62 | 5.64 | 42.59 | <0.01 | 4.38 | 5.37 | 36.91 | <0.01 |  |
| s(Age):Sex | 2.58 | 3.23 | 6.54 | 0.10 | 2.56 | 3.20 | 6.58 | 0.10 |  |
| s(FAM_ID) |  |  |  |  | 0.99 | 1.00 | 104.94 | <2e-16 |  |
| **DYS** | **Estimate** | **SE** | **z** | **P** | **Estimate** | **SE** | **z** | **P** |  |
| (Intercept) | -4.80 | 0.05 | -105.24 | <2e-16 | -4.52 | 0.07 | -65.39 | <2e-16 |  |
| Sex(Female) | 0.52 | 0.05 | 9.53 | <2e-16 | 0.52 | 0.05 | 9.57 | <0.01 |  |
| **Smooth function** | **edf** | **Ref.df** | **Chi.sq** | **P** | **edf** | **Ref.df** | **Chi.sq** | **P** |  |
| s(Age) | 4.49 | 5.52 | 44.30 | <0.01 | 4.54 | 5.57 | 39.72 | <0.01 |  |
| s(Age):Sex | 1.00 | 1.00 | 0.89 | 0.35 | 1.00 | 1.00 | 0.91 | 0.34 |  |
| s(FAM_ID) |  |  |  |  | 0.96 | 1.00 | 26.43 | <0.01 |  |
| **GAD** | **Estimate** | **SE** | **z** | **P** | **Estimate** | **SE** | **z** | **P** |  |
| (Intercept) | -3.48 | 0.02 | -142.50 | <2e-16 | -3.18 | 0.04 | -86.41 | <2e-16 |  |
| Sex(Female) | 0.53 | 0.03 | 18.19 | <2e-16 | 0.53 | 0.03 | 18.25 | <2e-16 |  |
| **Smooth function** | **edf** | **Ref.df** | **Chi.sq** | **P** | **edf** | **Ref.df** | **Chi.sq** | **P** |  |
| s(Age) | 5.75 | 6.80 | 148.85 | <2e-16 | 5.63 | 6.68 | 136.54 | <2e-16 |  |
| s(Age):Sex | 2.53 | 3.17 | 4.32 | 0.23 | 2.62 | 3.29 | 4.71 | 0.20 |  |
| s(FAM_ID) |  |  |  |  | 0.99 | 1.00 | 113.65 | <2e-16 |  |
| **PD** | **Estimate** | **SE** | **z** | **P** | **Estimate** | **SE** | **z** | **P** |  |
| (Intercept) | -6.54 | 0.11 | -57.80 | <2e-16 | -6.43 | 0.15 | -44.16 | <2e-16 |  |
| Sex(Female) | 0.62 | 0.13 | 4.82 | <0.01 | 0.63 | 0.13 | 4.83 | <0.01 |  |
| **Smooth function** | **edf** | **Ref.df** | **Chi.sq** | **P** | **edf** | **Ref.df** | **Chi.sq** | **P** |  |
| s(Age) | 5.11 | 6.16 | 34.92 | <0.01 | 5.13 | 6.18 | 34.57 | <0.01 |  |
| s(Age):Sex | 1.20 | 1.36 | 1.55 | 0.24 | 1.22 | 1.39 | 1.53 | 0.24 |  |
| s(FAM_ID) |  |  |  |  | 0.58 | 1.00 | 1.39 | 0.12 |  |
| **SPH** | **Estimate** | **SE** | **t** | **P** | **Estimate** | **SE** | **z** | **P** |  |
| (Intercept) | -4.86 | 0.05 | -102.83 | <2e-16 | -4.71 | 0.07 | -63.18 | <2e-16 |  |
| Sex(Female) | 0.21 | 0.06 | 3.59 | <0.01 | 0.21 | 0.06 | 3.57 | <0.01 |  |
| **Smooth function** | **edf** | **Ref.df** | **Chi.sq** | **P** | **edf** | **Ref.df** | **Chi.sq** | **P** |  |
| s(Age) | 4.69 | 5.72 | 39.06 | <0.01 | 3.62 | 4.49 | 30.78 | <0.01 |  |
| s(Age):Sex | 2.28 | 2.86 | 1.66 | 0.67 | 3.71 | 4.59 | 5.19 | 0.27 |  |
| s(FAM_ID) |  |  |  |  | 0.87 | 1.00 | 6.97 | 0.01 |  |

*Chi.sq, Chi-squared test value; DYS, dysthymia; edf, effective degrees of freedom; GAD, generalized anxiety disorder; ID, identifier for each subject; MD, major depression; P, p-value; PD, panic disorder; edf, effective degrees of freedom; Ref.df, reference number of effective degrees of freedom used for hypothesis testing; SE, standard error; SPH, social phobia, z, z-test value.*

*Parametric and smooth terms of generalized additive models describing each internalizing disorder using age and sex as independent variables, and allowing a separate smooth for men and women. The models on the right contain an additional parameter accounting for the random effect of family membership. The reference class for sex are men. The ‘edf’ reflects the number of effective degrees of freedom, which is an estimate of how many parameters are needed to represent the smooth. An edf>1 indicates a nonlinear smooth. The P-value associated with the smooth term indicates if the smooth is significantly different from 0.(1) Although the intercepts of the GAMs were clearly significantly different across the internalizing disorders, the curves over age were similar for men and women, in models with and without the family membership term. In other words, the relative difference in prevalence between men and women remained stable across different ages even when family membership was accounted for.*
